# Streamlining the screening cascade for active Hepatitis C in Russia: A cost-effectiveness analysis

**DOI:** 10.1371/journal.pone.0219687

**Published:** 2019-07-16

**Authors:** Paul Jülicher, Vladimir P. Chulanov, Nikolay N. Pimenov, Ekaterina Chirkova, Anna Yankina, Claudio Galli

**Affiliations:** 1 Health Economics and Outcomes Research, Abbott Diagnostics, Wiesbaden, Germany; 2 Reference Center for Viral Hepatitis, Central Research Institute of Epidemiology, Moscow, Russia; 3 I.M. Sechenov First Moscow State Medical University, Moscow, Russia; 4 Medical Communication, Abbott Diagnostics, Khimki, Russia; 5 CIS, Moscow, Russia; 6 Global Medical & Scientific Affairs, Abbott Diagnostics, Rome, Italy; Duke University, UNITED STATES

## Abstract

**Objective:**

Screening for hepatitis C in Russia is a complex process that involves several visits and stepwise testing, limiting adherence and substantially reducing the yield in the identification of active infections. We aimed to evaluate the cost-effectiveness of different screening algorithms from a health system perspective.

**Methods:**

A decision analytic model was applied to a hypothetical adult population eligible to participate in a general screening program for hepatitis C in Russia. The standard pathway (I: Screen for anti-HCV antibodies followed by a nucleic acid test for HCV RNA on antibody positives) was compared to three alternatives (II: Screen for antibodies, a reflexed test for HCV antigen on antibody positives, and RNA on antigen negatives; III: Screen for antibodies, a reflexed test for HCV antigen on antibody positives; IV: Screen for antigen). Each strategy considered a cascade of events (referral, adherence, testing, diagnosis) that must occur for screening to be effective. The primary measure of effectiveness was the number of diagnosed active infections. Calculations followed a health system perspective with costs derived from 2017 reimbursement rates and a willingness-to-pay of 2,000RUB ($82) per diagnosed active infection. Model was tested with deterministic and probabilistic sensitivity analyses.

**Results:**

Non-adherence to screening stages reduced the capture rate of active infections in Strategy I from 79.0% to 40.6%. Strategies II, III, and IV were less affected and identified 69%, 67%, and 104% more infections. Average costs per diagnosed infection were decreased by 41% from 89,599RUB ($3,681) for I to 53,072RUB ($2,180), 53,004RUB ($2,177), and 59,633RUB ($2,450) for II, III, and IV, respectively. With a probability of 97%, Strategy III was most cost-effective with an incremental cost-effectiveness ratio vs. I of -1,373RUB (CI: -5,011RUB to -2,033RUB; $-56; CI: -$206 to -$84). Below a willingness-to-pay of 91,000RUB ($3,738), Strategy IV was not cost-effective. Sensitivity analyses confirmed the robustness of results.

**Conclusions:**

Testing strategies for hepatitis C with HCV antigen on HCV antibody positive cases offer a streamlining opportunity for population screening programs. Those shall increase the chances for detecting active infections and are cost-effective over current practice in Russia.

## Introduction

Globally, there are an estimated 71 million people living with chronic hepatitis C virus (HCV) infection [1]. With about 4.5 million infected subjects, Russia is ranked among the countries with the highest number of HCV infections worldwide [2, 3]. The total economic burden associated to HCV and its sequelae was estimated to be 48 billion Rubles ($1.98 billion) in the Russian Federation in 2010 [4]. The advent of highly tolerable direct acting antiviral agents (DAA) has brought promise to efficiently control the disease. In 2016, the World Health Organization (WHO) set hepatitis elimination goals which target an 80% decline in HCV incidence and a 65% reduction in HCV related mortality by 2030 [5]. Still, it is estimated that most people living with HCV are undiagnosed or unaware of their infection [6]. In addition, access to affordable hepatitis testing is limited, particularly in low- and middle-income countries [6]. Treatment update is still low leading to the situation that there were more new HCV infections than patients who were started on treatment in 2015 [1, 7]. To reach WHO targets, several countries are formulating HCV strategies aiming at the identification of active infections and to solve the issues and barriers alongside the cascade of care [8–10]. Screening activities must reach the higher proportion of undiagnosed people to have a major population-based impact [9]. Therefore, continuous efforts towards more effective screening strategies including a higher testing rate are required [6, 7, 10, 11]. Testing guidelines indicate the need to screen individuals for antibodies to HCV (AB) and to confirm the presence of viral replication [8, 12, 13]. The latter is achieved by testing for HCV-RNA by nucleic acid amplification methods (NAT) or for the HCV core antigen (AG). The latter, though less sensitive than current NAT methods, bears several operative advantages and guarantees an almost equivalent clinical sensitivity and has been recently emerged as a suitable alternative to RNA testing [6, 14–18]. Most approaches include several steps in the diagnostic process that often lead to incomplete diagnosis with a positive anti-HCV result not followed by confirmatory tests, that is an almost universal gap in HCV screening [11, 19]. According to the official guideline for hepatitis C screening in Russia [20], people are referred to a laboratory for initial screening with AB. All reactive samples must be confirmed in the laboratory in duplicates and a subsequent AB before sending results back to the referring physician. In case of a positive AB status, individuals are sent to an infectious disease specialist or a gastroenterologist. In case confirmation of active infection is required, the expert refers people to a laboratory for RNA testing. The final diagnosis of an active HCV infection is made on an expert level based on screening results and after conducting a comprehensive clinical and laboratory examination. It is important to note that the current cascade of testing in Russia requires people to go to different places in order to get diagnostically conclusive results. Therefore, the number of complete diagnosis and the effectiveness of screening massively relies on people’s adherence to medical referrals. Since a loss to follow-up occurs at each step of the process, the need to consolidate the screening cascade has become apparent [11, 19, 21]. Suggested simplified algorithms, and even a one-step diagnostic procedure, have been proposed [17, 22], but in most instances a sound health economic evaluation is lacking, since studies are usually focused on direct costs only [15, 18]. The objective of this study was therefore to evaluate the cost-effectiveness of different screening algorithms for identifying patients with active hepatitis C infections while considering implications on the screening cascade in Russia.

## Methods

### Basic model structure

**Fig 1:**
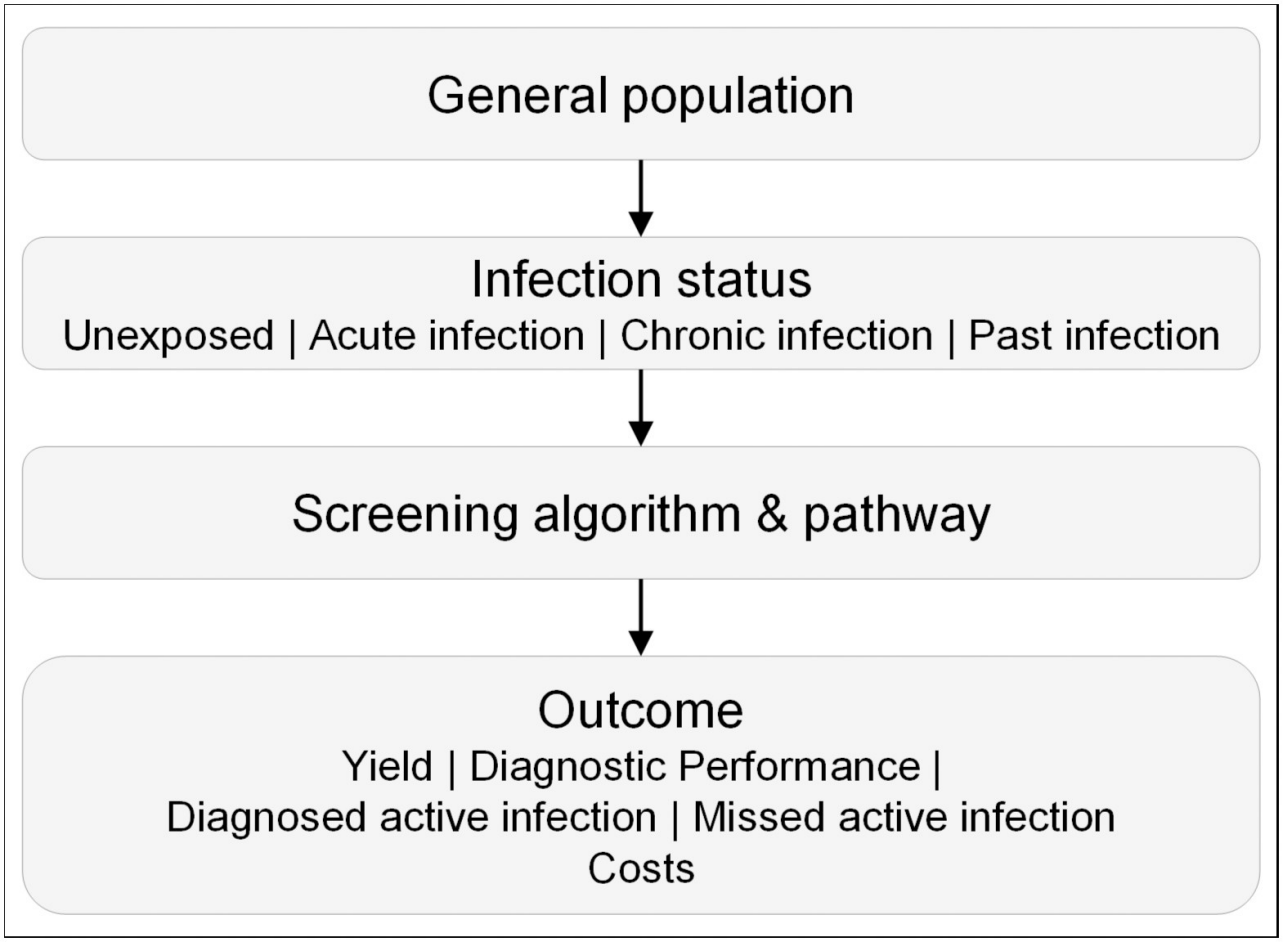
Basic model structure.

We modified a recently published decision analytic model to consider drop-offs within the cascade of screening [23]. The model was applied to a hypothetical adult population, who were eligible to participate in a general screening program for Hepatitis C in Russia. The study focused on the patient journey through the screening program from a health system perspective with an immediate time horizon. The basic structure and flow is depicted in Fig 1. Individuals entered the model in an asymptomatic and prior unknown health status related to HCV infection that encompassed four clinical conditions: no infection, acute HCV infection (AHC), chronic HCV infection (CHC), and past infection, the latter being characterized by the lack of markers of viral replication in the presence of specific anti-HCV antibodies. The model followed individuals through different pathways employing alternative algorithms. Given the result of the algorithm, individuals were finally categorized as true positive, true negative, false negative, or false positive referring to the status of an active HCV infection. An active infection is defined as the presence of markers of viral replication in either an acute or chronic infection state. Subjects with true-positive results were counted as ‘diagnosed active HCV infections’(DAI); those with false-negative results remained undiagnosed and were counted as ‘missed active infections’ (MAI). The model also counted individuals who did not adhere to the requested pathway and were lost at a certain stage in the screening cascade. The primary outcome measure of effectiveness was the number of diagnosed active infections. Strategies were compared in terms of the total costs of screening and diagnosing an active HCV infection.

### Standard and alternative strategies

The model compared four strategies based on distinctive screening algorithms consisting of a serological test for antibodies (AB), a nucleic acid test for HCV RNA (RNA), and a serological test for HCVAg (AG). Each strategy considered a cascade of events including referral, adherence, testing, and diagnosis that must occur for screening to be effective.

**Fig 2:**
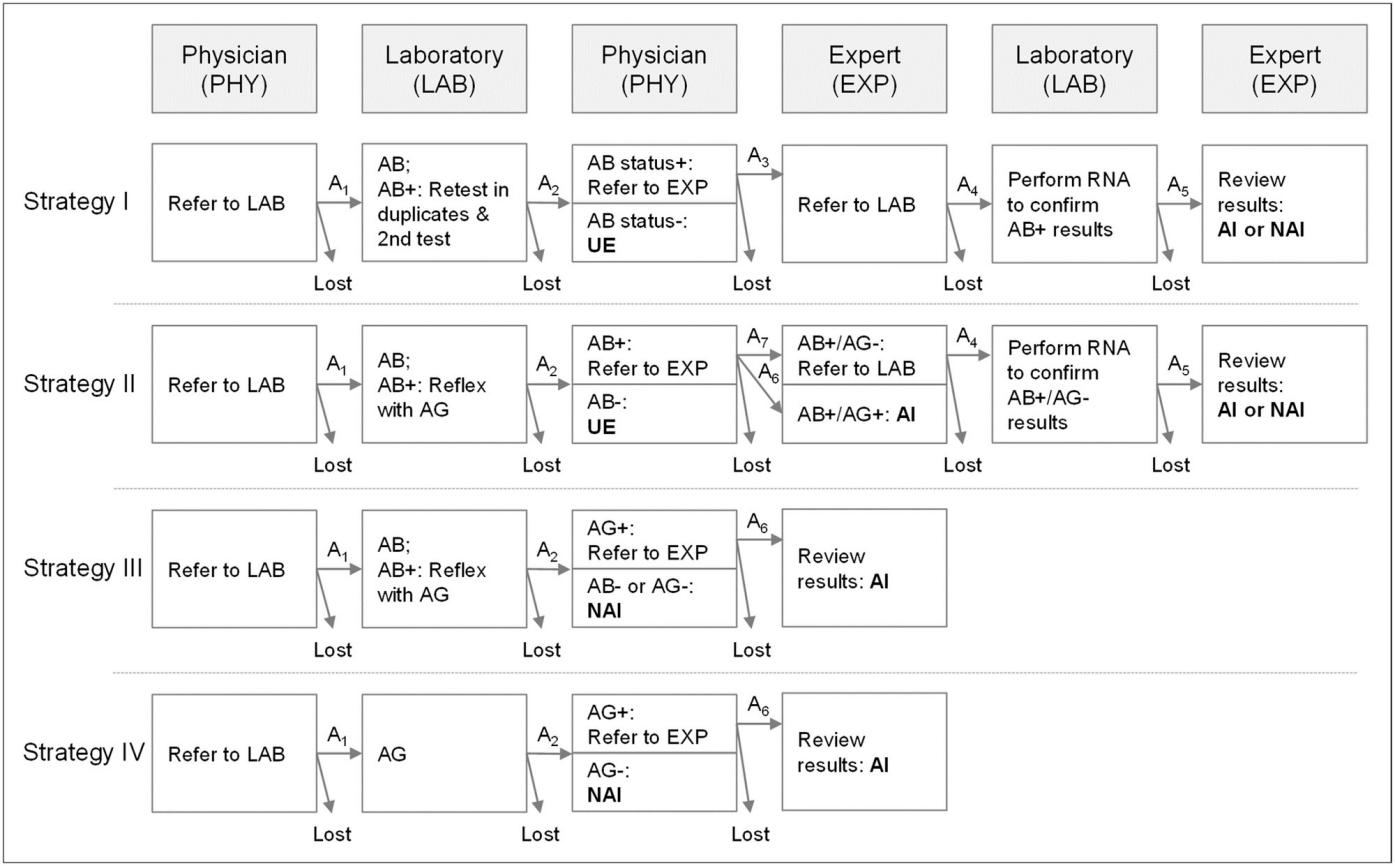
Standard and alternative HCV screening cascades of care. UE: unexposed; AI: Active HCV infection; NAI: No active HCV infection. AB: HCV antibody test; AG: HCV antigen test; RNA: HCV nucleic acid test. A_1-7_: Adherence rates.

In current practice, people are initially screened with AB, and reactive samples are confirmed in the laboratory in duplicates and a subsequent AB [20]. The presence of active infections is confirmed by testing on RNA. Referrals to the laboratory for testing rely on the decision of a general practitioner, an infectious disease specialist or a gastroenterologist. The final diagnosis of an active HCV infection (AHC or CHC) is made on an expert level based on screening results and after conducting a comprehensive clinical and laboratory examination. The standard pathway is described as Strategy I in Fig 2. Like Strategy I, AB was used as the front-line test for screening in Strategy II and III. Reactive AB samples were however reflexed with testing for AG. Samples with AB+/AG+ were regarded as “confirmed active infection” (AI). In Strategy II, AB+/AG- samples required subsequent confirmation with RNA, whereas in Strategy III these results were interpreted as “no active infection” with no further follow-up. Strategy IV was based on a single marker screening strategy with AG. Genotyping and other pre-treatment examinations were assigned to all subjects who were diagnosed with an active HCV infection at the final expert level.

### Data sources and input assumptions

Input assumptions are shown in Tables 1 and 2. Hepatitis C seroprevalence, viremic rate, and prevalence rate of acute infection was estimated based on epidemiological studies from Russia [3, 17, 24, 25]. Viremic rate refers to the proportion of chronic infected individuals out of all seropositive cases. Prevalence for chronic and past HCV infection were derived from these data by multiplying the seroprevalence with the viremic rate, and by subtracting the prevalence for chronic infections from the seroprevalence, respectively. For assay performances, the RNA test was regarded as reference, with sensitivity and specificity assumed to be almost perfect. Assumptions for AB and AG performance distinguished between acute and chronic infections, considering a delayed antibody seroconversion in acute infections, and the probability of low level viraemia in a small proportion of subjects with chronic HCV [26]. Specificity and sensitivity information in CHC were derived from systematic reviews [16, 27], estimates for performance in AHC were used as reported in the literature [18]. Since no other information exists, a baseline adherence rate of 95% was assumed for steps in the cascade of care during screening (Fig 2: A_1_, A_2_, A_5_). Data from a Russian registry showed that only 60% of people with a positive antibody result were tested with RNA (Fig A in S1 File). Based on standard practice, this process can be divided into four subsequent steps: 1. adherence of patients to follow the referral to an expert; 2. prescription to laboratory for confirmation; 3. adherence of the patient to visit the laboratory; 4. testing. The model considered these chances by two adherence rates: the adherence to visit the expert A_3_, and the adherence to visit the lab A_4_ (Fig 2). Given the fact that patients were referred to the expert in case of a positive antibody status only, a self-awareness of being positive was supposed which was linked to a reduced motivation to follow the recommendation for getting final confirmation. In addition, a positive selection of motivation was assumed during the screening cascade, meaning that some patients followed the pathway under any circumstances which ultimately increased the adherence rate on subsequent steps. Therefore, we assigned adherence rates of 75% and 80% to A_3_ and A_4_, respectively, which would finally match to the 60% as observed in the registry. In Strategy II and III, positive antibody samples were directly reflexed with AG in the laboratory with a positive AG result being indicative for a confirmed active infection. The same status applied to AG positive cases in Strategy IV. We assumed a higher motivation and improved adherence rate for patients in this state to seek potential cure by visiting a specialist and set the adherence rate A_6_ to the standard value of 95% (Fig 2). Patients with a AB+/AG- finding in Strategy II required subsequent confirmation with RNA. Those patients were assumed to have a negative self-awareness regarding an infection, meaning that a negative second test result led to a perception of low-risk. Thus, a substantially lowered motivation to follow the referral appeared reasonable. Therefore, we set the adherence rate A_7_ to 50%. Total yield referred to the proportion of subjects with complete diagnosis which implies subjects who followed all required elements of the respective screening cascade. All adherence estimates were tested across a ±10% range in univariate sensitivity analyses. In addition, calculations were also done by assuming a perfect scenario for all steps. Costs were calculated in accordance with the official reimbursement rates for payment of medical care in Russia [28]. Costs represent unweighted average values in 2017 Rubles from selected regions in each of the eight Russian federal districts. Regions were selected on availability and consistency of required information and are presented in Table B in S1 File. Since no reimbursement exists for AG, a mean was estimated being approximately half of the average reimbursement rate for RNA. Ranges used in sensitivity analyses (SA) reflected the minimum and maximum reimbursement across all regions. In the probabilistic sensitivity analysis (PSA), average rates were estimated by randomly sampling a value between the first and third quartile of the respective LogNormal distribution to account for weighting uncertainty. Costs were not discounted given the short-term horizon of the screening pathway. Cost-effectiveness calculations were assuming a willingness-to -pay threshold (WTP) of 2,000 Rubles ($82) per additional identified active HCV infection. No accurate information on WTP in this context exists. Therefore, our estimate was informed by a formerly required out-of-pocket-payment for performing a nucleic acid test for RNA. For this we used the 75^th^ percentile of RNA reimbursement rates from the Russian regions selected. The conversion from Russian Rubel to US dollar (100 RUB = $4.11) was based on 2017 OECD purchasing power parities [29].

**Table 1:** General input assumptions.

Variable	Base case	(SD)	Distribution	SA Range	References
Population					
HCV antibody positive	0.041	0.002	Beta	0.035–0.046	[3, 24]
Viremic rate	0.71	0.01	Beta	0.40–0.81	[3, 25]
Acute hepatitis C (AHC), per 100,000	1.52	0.03	Beta	1.0–2.0	[17, 24]
Adherence rate (Yield)					
Lab visit for screening (A_1_)	0.95	0.02	Beta	0.86–1.00^1^	Assumption
Revisit physician (A_2_)	0.95	0.02	Beta	0.86–1.00^1^	Assumption
Expert visit (A_3_; S1: Ab+)	0.75	0.02	Beta	0.68–0.83^1^	Registry
Expert visit (A_6_; S2&S3: Ab+/ Ag+; S4: AG+)	0.95	0.02	Beta	0.86–1.00^1^	Assumption
Expert visit (A_7_; S2: Ab+/Ag-)	0.50	0.02	Beta	0.45–0.55^1^	Assumption
Expert visit (A_5_)	0.95	0.02	Beta	0.86–1.00^1^	Assumption
Lab visit for confirmation (A_4_)	0.80	0.02	Beta	0.72–0.88^1^	Registry
Test performance					
AB sensitivity in AHC	0.600	0.003	Beta	0.450–0.750	[18]
AB sensitivity in CHC	0.818	0.003	Beta	0.590–1.000	[27]
AB specificity	0.997	0.001	Beta	0.975–0.997	[27]
AG sensitivity in AHC	0.999	0.001	Beta	0.950–0.999	[18]
AG sensitivity in CHC	0.9672	0.001	Beta	0.964–0.970	[16]
AG specificity	0.990	0.001	Beta	0.987–0.993	[16]
RNA sensitivity	0.999	0.0005	Beta	0.995–0.999	Assumption
RNA specificity	0.999	0.0005	Beta	0.995–0.999	Assumption

SA: Range tested in sensitivity analyses.

^1^ SA range estimated from base case value ±10%. Adherence rates A_1-7_ as illustrated in Fig 2. S1: Strategy I; S2: Strategy II; S3: Strategy III; S4: Strategy IV.

**Table 2:** Input assumptions for costs.

Variable	Average	Median	Q1—Q3	Distribution	SA Range
Visit to physician or expert	338	337	245–433	LogNormal	236–449
Registration, blood draw & sample processing in the lab	123	105	74–190	LogNormal	44–232
HCV Ab testing	252	158	101–405	LogNormal	89–705
HCV Ag*	600	570		LogNormal	250–2,000
HCV RNA test	1,339	1,162	342–2,028	LogNormal	244–3,582
Clinical tests	1,028	812	455–1,169	LogNormal	357–2,837
HCV RNA genotyping	1,841	1,525	1,078–2,763	LogNormal	647–3,582
Ultrasound imaging	519	282	152–949	LogNormal	134–1,576

With the exception of HCVAg, costs were calculated in accordance with the official reimbursement rates for payment of medical care in eight Russian regions in 2017 Ruble (100 Rubles = $4.11) [28]. Costs are unweighted average values. PSA sampling between Q1 and Q3 following a LogNormal distribution to reflect weighting uncertainty. Sensitivity analyses (SA) range reflects the minimum and maximum tariff in the considered regions.

*Mean reimbursement for HCVAG estimated from 50% of the average reimbursement rate for HCV RNA.

### Model calculations and statistics

The model was developed in TreeAge Pro 2018 (TreeAge Software, Williamstown, MA, USA). Statistical analyses were done in Minitab 17.1.0. (Minitap, Ltd. Coventry, UK). In order to assess the model’s accuracy for making relevant and meaningful predictions, a set of validation steps had been conducted [30]. For face validation, experts were reviewing model structure, inputs assumptions, data sources and results. Model structure was informed by a review of health economic modelling methodologies for HCV screening [31], and was designed to function as a front-end tool for a dynamic transmission model for epidemiological projections. Mathematical calculations were validated internally. Comparisons between strategies were made based on the respective means, the median values, and 95% confidence intervals calculated from the probabilistic sensitivity analysis (PSA). We obtained confidence limits for costs per diagnosed infection (CPDI), incremental cost effectiveness ratio (ICER), and incremental net monetary benefit (INMB) from the 2.5^th^ and 97.5^th^ percentile of the respective result distribution. Statistical significance was analyzed conducting a 2-Sample t-test with a significance level of 0.05 on average results from a PSA. The probability for cost-effectiveness of strategies were tested within a WTP range from 0 to 100,000 Rubles ($0 to $4,108) and presented as cost-effectiveness acceptability curves. Besides comparisons on deterministic results of the base case scenario, sensitivity analyses were conducted to consider parameter variability and to check the impact of individual variable uncertainty on results and the overall model robustness. During PSA, values were varied widely within the ranges stated in Tables 1 and 2, and were randomly sampled from the respective distributions with 5,000 iterations. All variables were assumed to be independent. Univariate sensitivity analyses were performed on all variables and were reported as incremental cost-effectiveness tornado diagrams. To evaluate thresholds that indicate a change in the preferred strategy, bivariate analyses were performed over a wide range for adherence rates, WTP, AG reimbursement rates, AG sensitivity, AB specificity, and AB sensitivity. In analyses that were reported as net monetary benefits (NMB), the effectiveness (Number of diagnosed active infections) were converted into monetary units by multiplying it with the WTP threshold, then subtracting the costs of the strategy. The strategy with the highest NMB was regarded as preferred choice [32]. The reporting of this study was done in accordance to the Consolidated Health Economic Evaluation Reporting Standards (CHEERS) [33].

## Results

**Fig 3:**
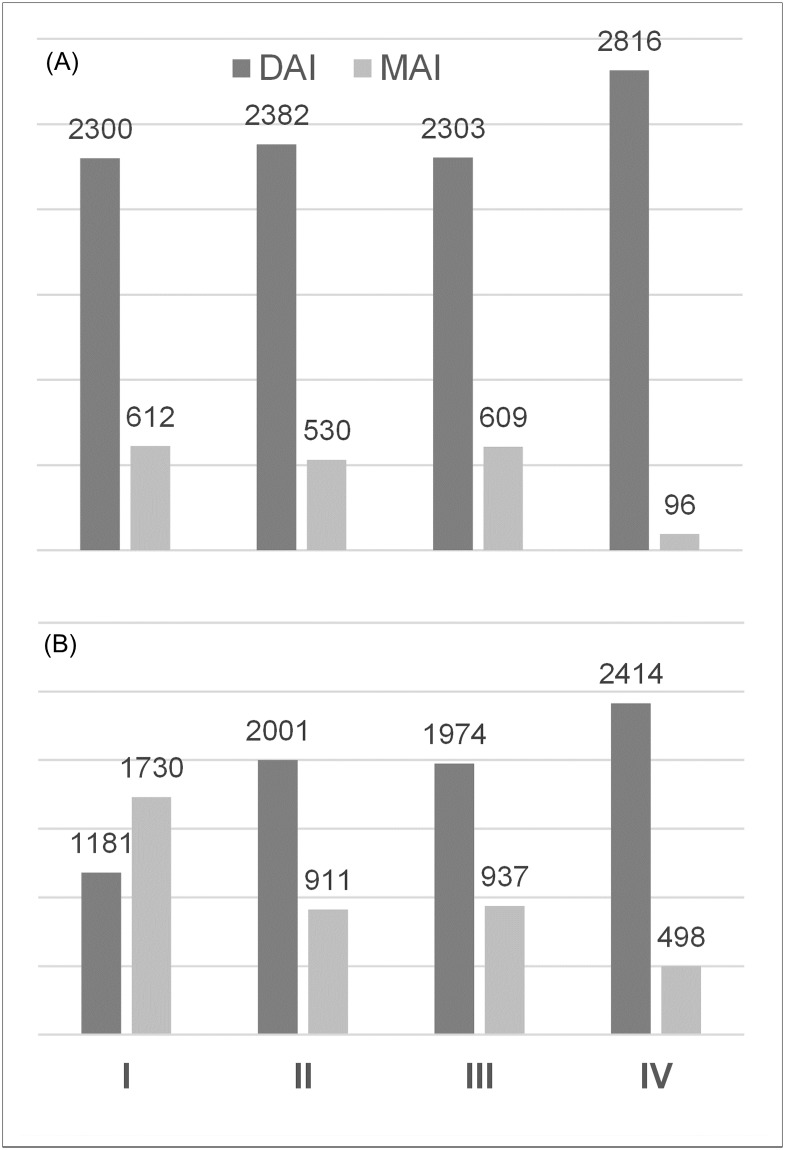
Impact of adherence on screening pathway effectiveness. Number of diagnosed active infections (DAI) and missed active infections (MAI) per 100,000 subjects screened. (A) Perfect adherence scenario. (B) Impaired adherence scenario. Monte Carlo simulation results with 5,000 iterations for each scenario assuming a prevalence of active HCV infections of 2.9%.

A comparison of deterministic and probabilistic results from Monte Carlo simulations with 5,000 iterations (PSA) showed good concordance and robust model results (Table D and E in S1 File). Hereinafter, except for deterministic sensitivity analyses, all results refer to outputs from the PSA. In a perfect adherence scenario, more active infections were identified with Strategy II and IV compared to current practice (Strategy I) resulting in a superior capture rate (II: 81.8% vs. 79.0%, p = 0.007; IV: 96.7%, p<0.001, Table 3). The capture rate of active infections with Strategy III was not significantly different compared to Strategy I with a slightly reduced diagnostic accuracy. In contrast, diagnostic accuracy for Strategy II (99.46%) and IV (98.96%) were significantly higher and lower compared to I (99.39%, p<0.001 for both). Although capturing more infections, the latter was caused by a substantial increase in false positive cases in Strategy IV (Fig B. in S1 File). Poor adherence to the requested screening cascade led to substantially reduced the overall number of complete diagnosis which is the total yield of subjects following the entire pathway (Strategy I: 89.0%; II: 89.4%; III: 90.1%; IV: 90.1%, Table 3). Accordingly, the capture rate of active HCV infections in Strategy I was impaired by 48% from 79.0% to 40.6%. The effectiveness’ of alternatives strategies were affected to a lesser extent: a loss of subjects during the screening cascade reduced the capture rate of active infections by 16.0%, 14.3%, and 14.3% for strategies II, III, and IV, respectively. Consequently, Strategy II, III, and IV identified 69%, 67%, and 104% more active infections compared to the standard, which was on average equal to 820 (II; 95%CI: 815 to 824), 793 (III; 95%CI: 789 to 797), and 1,233 (IV; 95%CI: 1,228. to 1,238) additionally detected active HCV infections for 100,000 subjects screened (Fig 3, Table 3).

**Table 3:** Impact of adherence on capture rate of diagnosed infections.

	Perfect adherence				Impaired adherence			
	Diagnostic Accuracy, %		Capture Rate, %		Total yield, %		Capture Rate, %	
	Mean	Diff. vs. I	Mean	Diff. vs. I	Mean	Diff. vs. I	Mean	Diff. vs. I
Strategy I	99.39		79.0		88.95		40.6	
Strategy II	99.46	0.07*	81.8	2.8*	89.37	0.41*	68.7	28.2*
Strategy III	99.38	-0.01*	79.1	0.1	90.11	1.16*	67.8	27.2*
Strategy IV	98.96	-0.42*	96.7	17.7*	90.05	1.09*	82.9	42.3*

Results of each scenario from Monte Carlo simulations with 5,000 iterations. Yield refers to the proportion of complete diagnosis according to the screening protocol. The perfect adherence scenario equals a yield of 100%. Capture rate refers to the proportion of diagnosed infections divided by the total number of active infections.

*Significance test p-value ≤ 0.001.

In the impaired adherence scenario, total costs for screening were slightly higher with Strategy II (1,058 vs. 1,053 RUB per subject screened or $43.26 vs $43.46; 95%CI of difference: 1 to 9 RUB; Table 4) compared to Strategy I, but reduced with Strategy III (1,043 vs. 1,053 RUB or $42.85 vs. $43.26; 95%CI of difference: -15 to -7 RUB). In contrast, substantially increased costs were observed for Strategy IV (1,435 vs. 1,053 RUB or $58.95 vs. $43.26; 95%CI of difference: 370 to 382 RUB). The average costs per diagnosed infection (CPDI) were decreased by 34 to 41% from 89,599 RUB ($3,681; CI: 71,435–112,349 RUB) for Strategy I to 53,072 RUB ($2,180; CI: 43,211–65,101 RUB), 53,004 RUB ($2,177; CI: 43,035–65,067 RUB), and 59,633 RUB ($2,450; CI: 45,682–79,867 RUB) for Strategy II, III, and IV, respectively. Assuming a WTP threshold per diagnosed infection of 2,000 Rubles ($82), Strategy III was most cost-effective. The incremental cost-effectiveness ratio (ICER) was estimated to be -1,373 RUB (-$56.40; CI: -5,011; 2033 RUB) with a probability of being preferred (both cost-saving and cost-effective) compared to Strategy I of 97.3%. Strategy III dominated the current practice (Incremental DAI >0; Incremental costs < 0; Table 4) in 77% of all runs during probabilistic sensitivity analysis. While Strategy II and IV were found more effective, confidence intervals for ICERs relative to the next best alternatives appeared between 13,678 and 201,226 Rubles ($562 –$11,964) which were much higher than the assumed WTP (Table 4). Not considering all other alternatives, the probability of Strategy II and IV for being cost-effective compared to I with an ICER below the WTP threshold was 81% and 2%. Compared to the next best Strategy, II and IV were found cost-effective in 0% and 1% of cases, respectively (Table G in S1 File). Cost-effectiveness results for the perfect adherence scenario are provided in the supplement (Table F in S1 File).

**Table 4:** Cost-effectiveness results.

Strategy	Costs, RUB			Diagnosed active infections (DAI)			CPDI		ICER	
	Mean	Incr.	Incr. 95%CI	Mean	Incr.	Incre. 95%CI	Mean	95%CI*	Mean	95%CI*
Strategy I	1,053			1,181			89,599	(71,435; 112,349)		
**Strategy III**	**1,043**	**-11**	**(-15; -7)**	**1,974**	**793**	**(789; 797)**	**53,072**	**(43,211; 65,101)**	**-1,373**	**(-5,011; 2,033)**
Strategy II	1,058	16	(12; 19)	2,001	27	(22; 32)	53,004	(43,035; 65,067)	58,227	(40,541; 80,505)
Strategy IV	1,435	376	(370; 382)	2,414	413	(407; 419)	59,633	(45,682; 79,867)	91,463	(13,678; 201,226)

Results from Monte Carlo simulation with 5,000 iterations (PSA). List sorted by increasing number of diagnosed active infections. All costs in 2017 Rubles (100 RUB = $4.11). DAI: Diagnosed active HCV infection per 100,000 screened subjects. CPDI: Costs per diagnosed active infection. ICER: Incremental cost-effectiveness ratio; incremental costs divided by incremental DAI. CPDI and ICER confidence intervals estimated from the 2.5^th^ and 97.5^th^ percentile of the respective result distributions. The most cost-effective strategy at a WTP threshold of 2,000 Rubles ($82) per additional detected infection is shown in bold. For results converted to US$ see Table H in S1 File.

**Fig 4:**
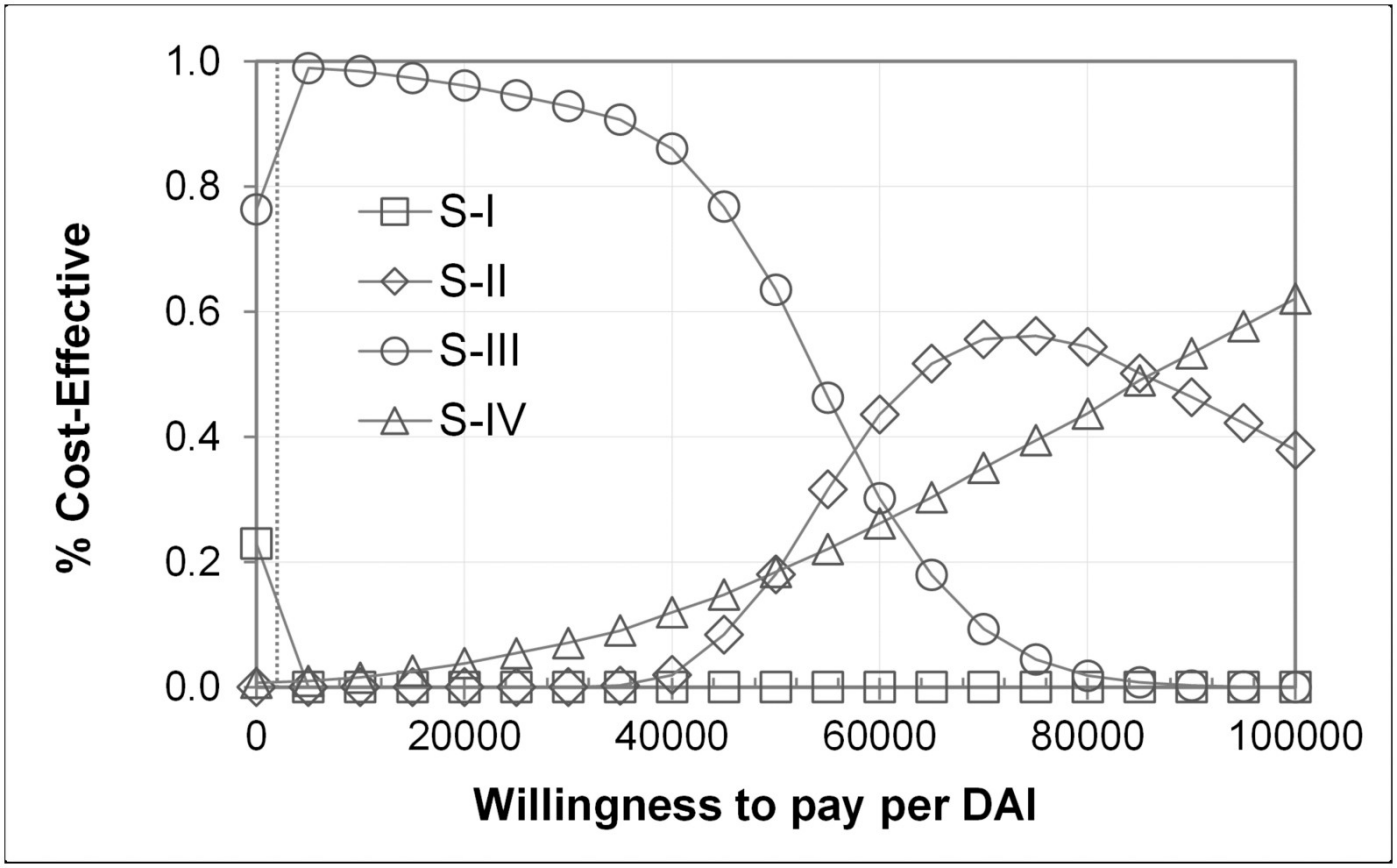
Cost effectiveness acceptability curves. Probability of cost-effectiveness of screening strategies as a function of the willingness to pay (WTP) threshold for one additional diagnosed active infection (DAI). WTP in 2017 Rubles (100 RUB = $4.11). The dotted line refers to a WTP of 2,000 RUB ($82). S-I: Strategy I; S-II: Strategy II; S-III: Strategy III; S-IV: Strategy IV.

**Fig 5:**
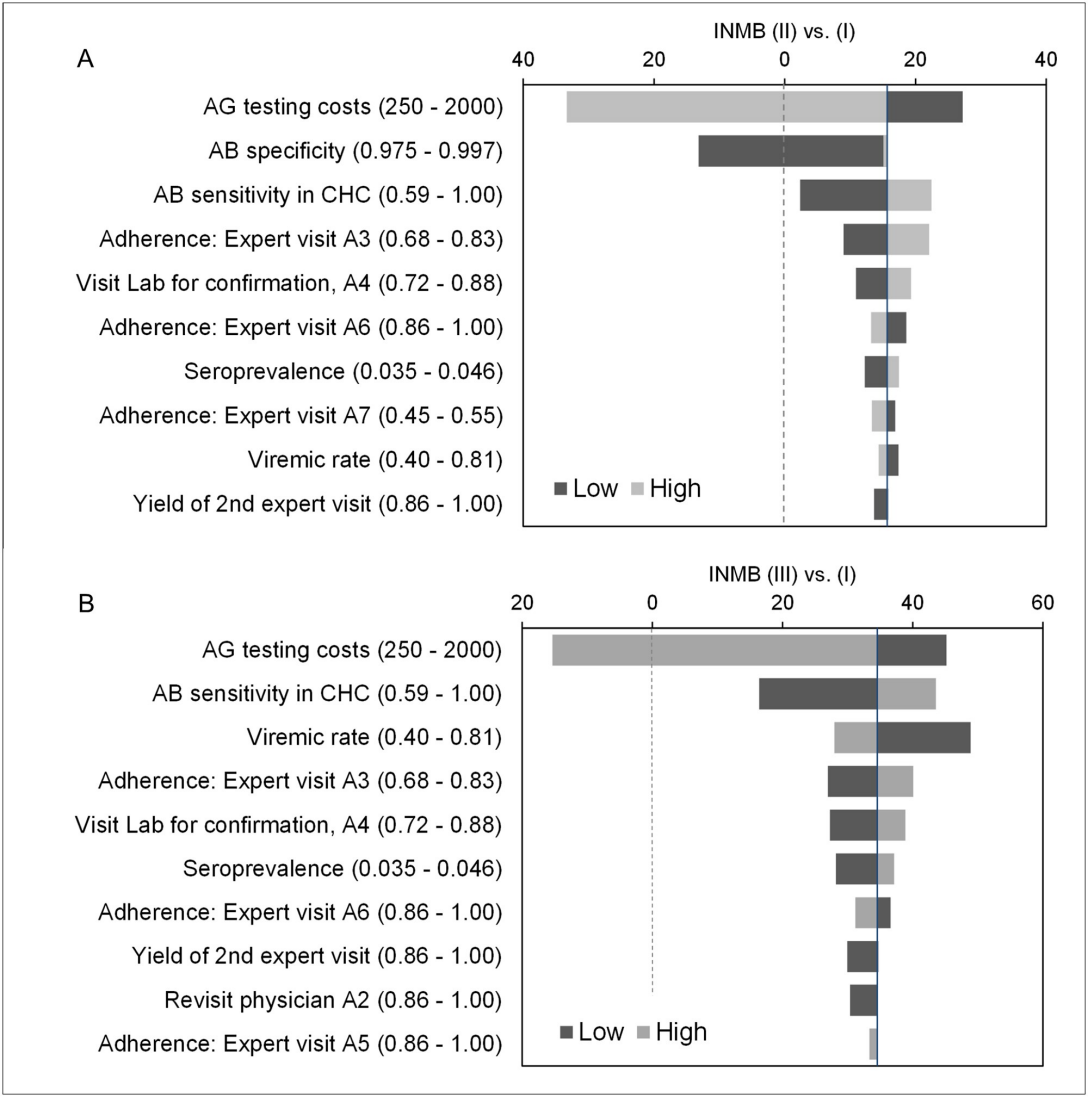
One-way sensitivity analyses tornado diagrams. Tornado diagrams from multiple one-way sensitivity analyses on all variables within the ranges stated in brackets. A: Incremental net monetary benefit (INMB) for Strategy II vs. Strategy I. B: INMB for Strategy III vs. Strategy I. NMB calculations based on WTP of 2,000 Rubles ($82). The vertical line represents the expected values using each variable’s base case value. The dotted line indicates a change of the preferred strategy within the range tested. Variables not presented here did not show an effect.

Cost-effectiveness-acceptability curves (Fig 4) show that Strategy III remained the preferred strategy with ≥ 90% probability between WTP thresholds ranging from 1,000 to 35,000 Rubles ($41 to $1,438). Strategy II and IV became the most cost-effective strategy at weights on the effect above 65,000 Rubles ($2,670) and 90,000 Rubles ($3,697), respectively. Incremental cost-effectiveness scatter plots from PSA are provided in the supplement (Fig C in S1 File). Tornado diagrams constructed from multiple univariate deterministic sensitivity analyses indicate which parameter uncertainty had the largest impact on model results. Within the tested ranges (SA range in Tables 1 and 2), incremental results were most sensitive to changes in AG testing costs, and AB specificity (Fig 5; Fig D in S1 File). For AG testing costs, a maximum value of 1,036 and 1,556 Rubles ($42.56 and $63.92) were calculated for Strategy II and Strategy III to be cost-effective compared to the standard. While Strategy-III remained the most cost-effective choice across the tested ranges for AB specificity and sensitivity, AB specificity below 98.6% favored the standard over Strategy II (Fig I in S1 File). At the base case WTP of 2000 RUB ($82), Strategy II remained preferred over I down to a threshold for AB sensitivity in CHC of 54.6%.

**Fig 6:**
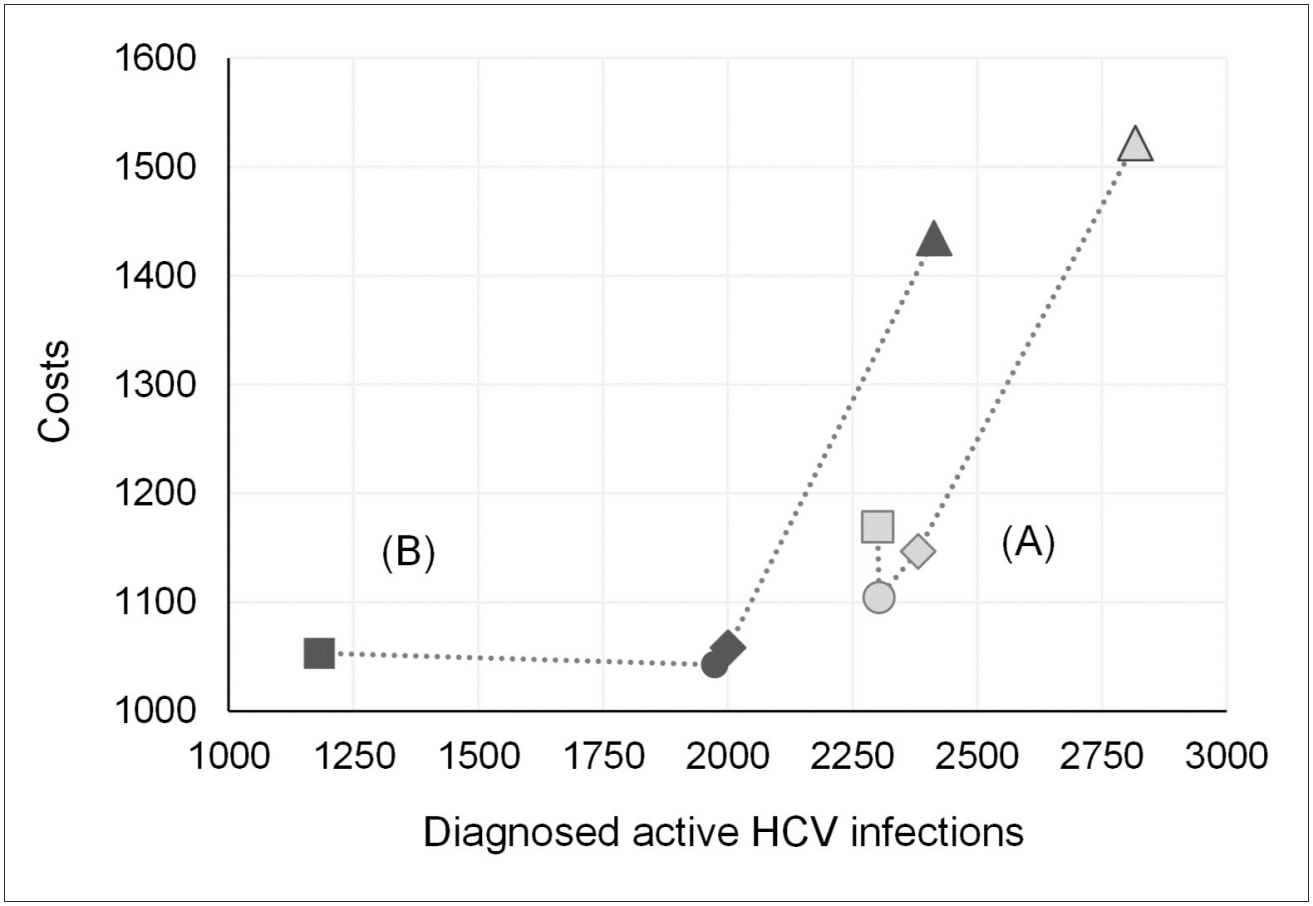
Cost-effectiveness plane. Average total cost vs. average diagnosed active infections (DAI) derived from a PSA with 5,000 iterations. Costs in 2017 Rubles (100 RUB = $4.11). DAI refers to 100,000 subjects screened. Perfect adherence scenario (A). Impaired adherence scenario (B). ■ Strategy I; ◆ Strategy II; ● Strategy III; ▲ Strategy IV.

A comparison of a perfect and impaired adherence scenario in a cost-effectiveness plane illustrates the impact of adherence on each of the strategies (Fig 6). While Strategy IV identified the most infected subjects, it also consumed considerably higher costs compared to AB based screening protocols. Overall, Strategy III remained cost-effective at a WTP of 2,000 Rubles ($82) in both scenarios by also dominating Strategy I. Focusing on strategies employing first-line AB screening, the standard strategy consumed the highest costs in a perfect adherence scenario. With subjects being lost due to impaired adherence, cost for Strategy I were also substantially reduced. Bottom-line, impaired adherence led to a 75.7% increase in the average costs per diagnosed infection (CPDI) for Strategy I, whereas Strategy II, III, and IV were affected to a much lesser extent showing a cost ratio increase of 10.4%, 9.8%, and 9.7%, respectively.

**Fig 7:**
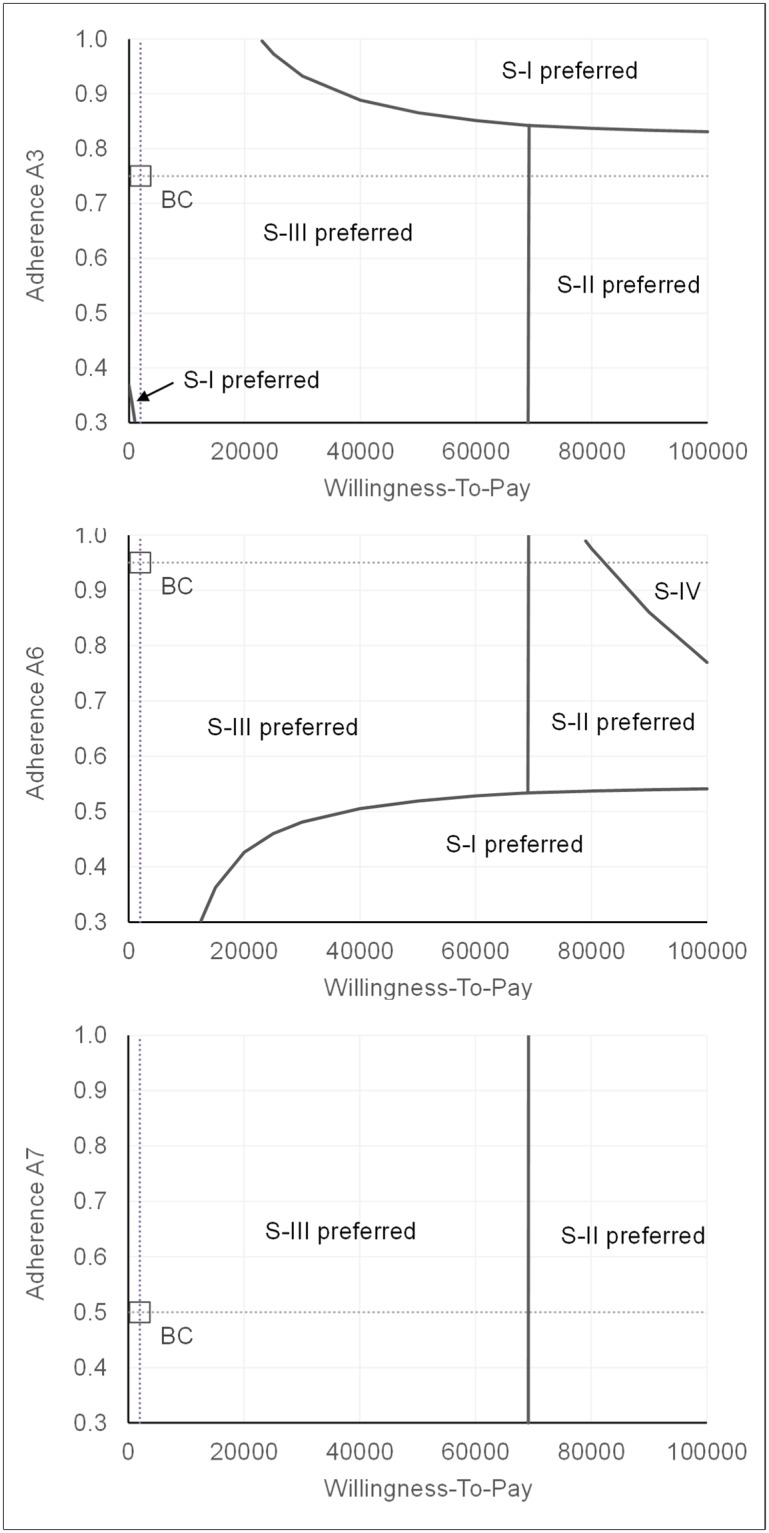
Threshold analysis for adherence rate assumptions. Bivariate threshold analysis of different adherence rates at variant willingness-to pay (WTP) thresholds for Strategy I to IV. WTP in 2017 Rubles (100 RUB = $4.11). The strategy with the highest net monetary benefit (NMB) was indicated as preferred option. BC: Base case assumption. S-I: Strategy I; S-II: Strategy II; S-III: Strategy III; S-IV: Strategy IV.

Bivariate threshold analyses demonstrate the influence of varying adherence rates on the preferred screening option while holding all other variables constant (Fig 7, Fig G in S1 File). A line in the graphs indicates thresholds of equivalence in terms of NMB between alternative strategies. In WTP range between 1,000 and 25,000 Rubles ($41 and $1,027), any adherence rate A3 favored Strategy III. At higher WTP and for adherence rates A3 greater than 83%, Strategy I remained preferred. WTP thresholds above 69,000 Rub ($2,835) changed the preference from Strategy III to the Strategy II. Adherence rates A6 between 30% and 100% had no impact on the ranking of strategies at a WTP of 2,000 Rubles ($82). In a range between 500 and 12,500 Rubles ($20 to $514), any value for A6 favored an alternative strategy. For A6 values greater than 54%, the alternate strategies were favored over current practice over the entire tested WTP range with a switch from Strategy III to the more effective strategies II and IV at WTP > 69,000 Rubles ($2,835). The standard Strategy I was found preferred in an area below an adherence rate A6 of 54% and above a WTP of 12,500 Rubles ($514).

Below WTP of 69,000 Rubles ($2,835), Strategy III remained the preferred option across the tested ranges for A7. Above that level, the preference switched to Strategy II. In summary, between a WTP of 1,100 and 12,000 ($45 and $493), Strategy III were preferred in terms of NMB at any value of A3, A6, and A7 in a range between 30% and 100%.

## Discussion

The cascade of care for HCV has been split into several steps [19, 34]. A substantial loss of patients between each step has been observed, thus pointing to challenges and barriers towards an efficient HCV care. Depending on countries, 27–56% of all infected patients reached the stage of a confirmed infection, and only 12–16% were finally referred to treatment [9, 11, 18, 34]. Against these challenges, some research has been made on how to increase engagement in HCV care to ensure that barriers are reduced. Several studies evaluated the effectiveness of different interventions to increase uptake of testing in different populations, or pointed to the need for optimizing the screening cascade [11, 34–36]. As well as all other interventions in health care, proposed new strategies in screening need undergo a framework of evidence generation with cost-effectiveness evaluations being an essential element [37]. Given the indirect impact of testing on patient outcome, engagement in the screening cascade as well as linkage to care are key drivers for cost-effectiveness of screening strategies [38]. Thus, suggested outcome measures in modeling studies should also consider the impact of screening interventions on uptake, case detection and linkage to care.

Several health economic evaluations of HCV screening were reported and evaluated in systematic reviews [31, 36, 38–40]. Most studies compared consequences of screening compared to no screening in specific risk groups or birth cohorts, and favorable cost-effectiveness was reported for targeted risk- or prevalence based screening strategies. It is important to note that evaluations of HCV screening mainly focused on consequences of guiding and managing individuals to and through treatment without a detailed or explicit view on the testing component of the model [31, 38–40]. While the uptake of treatment as the ultimate result of effective screening programs have been found considerably lower than assumptions used in cost-effectiveness studies of testing [36], explicit data on drop-offs within the cascade of testing have not been taken into account so far. Although most HCV screening models considered certain sequential screening pattern, a loss of subjects within the screening cascade was usually ignored. As an example, it is a common assumption that all AB-positive individuals would return for confirmation or follow the medical referral. One modelling study considered a loss to some extent by either considering a proportion of infected but undiagnosed subjects or by subsuming a potential loss to follow-up or to treatment in a single composite number [41]. In summary, the validity of modelling parameters of the testing phase was found difficult to assess, and the lack of details about the explicit cascade of testing, disregarded loss to follow-up between testing stages, or the use of composite, not assay-specific assumptions for sensitivity and specificity were supposed to overestimate modelling results [39]. To our knowledge, no cost-effectiveness study exists that explicitly focusses on completing the steps in-between the screening cascade such as from antibody positive findings to confirmed active infections. Therefore, we conducted a cost-effectiveness modeling study comparing three different alternative screening algorithms in terms of the number of diagnosed active HCV infections and costs to current practice for HCV screening. Since the current practice in Russia follows a logistically complicated multi-step screening process, we applied the model to this specific context and specifically took adherence between each of the steps into account. The alternative strategies in our study employed a hepatitis C antigen test (AG) that has been proven as an acceptable alternative to RNA for the detection of active HCV infections [13–16]. Against the need for more affordable HCV screening to control the disease [42, 43], the potential of AG to streamline the cascade of screening and to improve access to care has also been suggested [11, 16, 23, 44–46]. In our modelling study, we applied AG either in a direct reflex strategy in the laboratory on antibody positive samples or in a single marker screening strategy. In a first scenario, observed drop-off effects in the screening cascade were ignored (perfect adherence scenario), thus revealing the crude effectiveness of AG supported screening algorithms. Our analysis showed that all alternative strategies were at least as effective as the current practice. This is in line with previous findings that were demonstrating acceptable performance characteristics of AG and AG supported protocols [15, 23].

Considering a realistic degree of incomplete diagnosis in the analysis, however, reduced the capture rate of active infections in the standard strategy by almost half to around 41%. This dramatic drop in effectiveness is pointing to a substantial opportunity for improvement. Our study demonstrated that streamlining the screening cascade with AG increased the chances for detecting an active infection by up to 104%. In the context of this study this would translate to 793 to 1,233 more detected active infections per 100,000 subjects who underwent the first step of screening. Although statistically significant, the costs per screened subject were found in a comparable range between all strategies using AB first-line screening. The increased effectiveness of AG reflex strategies compared to current practice, however, led to a considerable improvement in efficiency. The costs per diagnosed active infection in both alternatives were reduced by 41% compared to current practice. Consequently, both AG reflex strategies were found cost-saving or cost-effective compared to current screening practice in Russia with a high probability of 81% or 98%. In contrast, using AG in a single marker screening strategy resulted in considerably higher costs mainly driven by an increased number of false positive screening findings. Although it would provide the most radical streamlining opportunity and superior capture rate, higher costs required for screening does not make the single marker screening with AG a cost-effective alternative in the context of reasonable willingness-to pay values in Russia. It should however be noted that the preference for one of the alternate strategies may also depend on aspects beyond willingness-to-pay and the scope of our study. This could be a higher weight on risk aversion to avoid missing infections as much as possible particularly in high risk groups. Also, barriers to implementation might become relevant drivers since a change in current practice usually requires substantial efforts. For example, although Strategy III demonstrated a preferred cost-effectiveness, Strategy II appears to require a lower level of change compared to the current situation and at the same time slightly reduces the risk of missing an infection.

As suggested by sensitivity analyses tornado diagrams (Fig 5, Fig D in S1 File), study results were most sensitive for varying assumptions in AB performance, and AG costs. The influence of changing AB performance on model results points to a critical issue in this area. Despite the global need to extend testing for HCV, systematic reviews on the diagnostic accuracy of common screening tests compared to RNA are surprisingly rare. Most studies focused on the performance of rapid tests compared to laboratory based methods [47, 48]. In a general population setting which is relevant to our study, one systematic review identified three studies that compared immunoassay performance relative to an RNA-based reference [27]. AB sensitivity ranged from 61.0% to 81.8% and specificity ranged from 97.5% to 99.7%, but overall quality of evidence was assessed to be low. Another review also reported a high heterogeneity in tests and population [39]. Quality of evidence was assessed low using the GRADE methodology [49]. Finally, the authors decided not to conduct a meta-analysis because of the paucity of evidence. Both reviews underlined some methodological challenges. For our study, there was a remaining level of uncertainty in the AB performance that we addressed in sensitivity analyses. In the base case scenario, AB sensitivity in CHC above 0.545 and AB specificity above 0.986 favored Strategy II over current practice (Fig I in S1 File). No threshold was observed for Strategy III, and this alternative appeared to be not affected by varying AB performance. In fact, policy-makers, government officials, and health care practitioners should be aware that the performance of different antibody tests available in specific regions may vary widely.

It is important to note that AG is still not covered in Russia and no reimbursement rate exist that could inform our analysis. Thus, we varied the input assumption over a wide range around the base case value from -58% to +230%. Consequently, rather than testing model sensitivity, results might be used for discussing an appropriate reimbursement range for AG. Cost-effectiveness thresholds for the alternative strategies with increasing willingness-to pay can be found in the supplement (Fig G in S1 File).

As demonstrated by one-way sensitivity analyses, model results were robust to all other variables including AG performance within the considered range. More details are provided in the supplemental file (Fig C to J in S1 File). We would like to underline that conclusions were informed by probabilistic sensitivity analysis (PSA) results. In PSA, all input values are varied simultaneously with values being sampled from a priori defined probability distributions [50]. Since these distributions consider uncertainty in input values over a reasonable range, confidence intervals derived from PSA actually reflect this level of variability, and in comparison to deterministic point estimates, they demonstrate the robustness to changes in model inputs.

Our study distinguishes acute, chronic and past infections and the distinct features of available diagnostic tools. Being the first-line test in the screening cascade, the performance of AB does play a vital role. While antibodies are detectable in most people with a chronic infection, in the acute phase the antibody response is usually delayed by several weeks [51]. In this setting an AG assay, although of inferior analytical sensitivity to RNA assays, detects acute HCV infection with similar clinical sensitivity [18, 44]. This benefit in sensitivity can be observed in Strategy IV as compared to all other strategies which are based on initial screening with AB.

The underlying assumption in most previous cost-effectiveness studies on HCV screening that all individuals tested positive with AB would proceed to RNA for diagnosing an active infection has been questioned as being unrealistic, and respective models are expected to overestimate both the costs and effectiveness of screening [39]. This statement is supported by our findings: The comparison of costs and effectiveness of all tested strategies in a perfect and an impaired adherence clearly indicates a considerable impact of adherence on cost-effectiveness outcomes. In conclusion, the screening component of screening models is traditionally poorly described and deserve explicit attention. The reasons for not completely engaging to the multistep cascade of test ordering, testing, and result reviewing that usually requires different visits to health care providers are complex. Besides lack of infrastructure and service availability, people’s motivation is driven by awareness, perception, education, and costs [21, 41, 43]. The use of HCV antigen tests has been suggested as a cheaper and simpler method for detecting viremia that may help to reduce some barriers by improving affordability, availability and accessibility of confirmation [16]. Although findings may not be generalized, a systematic review found that people expressed a desire for convenient testing in which sufficient information about HCV and testing is provided and results are obtained quickly [39]. One study demonstrated a positive effect of streamlining the HCV screening process. Switching from independent test ordering for confirmation to a combined reflex test order approach led to a remarkable improvement from 78% to more than 99% of patients with positive AB tested for RNA [21]. An automatic reflex test approach was also used in our study, and we showed that a direct reflex test with AG could eliminate the need for additional steps and visits within the cascade of screening. In the context of our study, a such streamlined cascade of screening increased the proportion of infected and detected subjects by 69% (Strategy II) or 67% (Strategy III). The patient’s willingness to adhere to the complete screening program has also been demonstrated in other medical areas as an ultimate driver for effectiveness [52]. The above cited systematic review provides a summary of studies dealing with people’s perceptions and preferences related to HCV screening [39]. The authors concluded that people make decisions while considering their immediate context and perception, and the availability of care. The willingness to pursue screening was found increased in people with a high perceived risk, and subjects with a low level of perceived risk stopped or rejected the process. In many cases, knowledge appears to have a positive effect on the acceptance of screening. Also, anxiety while waiting for test results were reported reasons for patients not to return for receiving results. It is noteworthy that elements that could provide advantages from improving adherence, reducing uncertainty in diagnostics, or the value of hope have been suggested in an extended approach to understand the value of new medical strategies [53]. Findings from the afore-mentioned studies were used to inform adherence rate assumptions for the alternative strategies in our model. Still in the specific context of our study, the reasons for non-adherence should be evaluated to better address patient’s concerns, loss to follow-up and associated waste of health care resources. Therefore, all described potential effects were subsumed in step specific probabilities, and were tested over a wide range in sensitivity analyses. Considering variability and uncertainty in adherence rates, the bivariate analyses clearly indicated that AG reflex strategies would remain cost-effective compared to current practice over a wide range of assumptions for adherence rates (Fig 7, Fig F in S1 File).

The willingness-to pay threshold is essential in health economic evaluations. The cost-effectiveness of interventions is traditionally evaluated against thresholds of cost-effectiveness using Quality-adjusted life years (QALYs) as a composite outcome measure of effectiveness [32, 53]. In our study, however, effectiveness of a strategy is described in terms of number of active HCV infections detected which has also been suggested as testing outcome in a recent meta-analysis of test interventions. Still, no definite cost-effectiveness threshold exists on this metric, and only few studies discussed the willingness-to-pay concept within this context [23, 31]. In the past, most Russian regions required out-of-pocket-payment for performing a nucleic acid test for RNA. Therefore, and in lack of other information, we used the RNA reimbursement rates as a base case assumption, and we tested this in sensitivity analyses across a wide range. Although identifying patients in an effective way has been recognized as a substantial driver for successful treatment pathways, still in many areas budgets for screening and treatment are isolated. Acknowledging that strategies were not always weighted against their effectiveness, we also considered the scenario of zero WTP which in fact reflects the cheapest strategy. As illustrated by the cost-acceptability curves (Fig 4), streamlined screening cascades provide a very high likelihood of being cost-effective compared to current practice over the entire tested WTP range from 0 to 100,000 Rubles ($0 to $4,108).

We would like to emphasize specific health policy implications of our study. First, our study clearly demonstrates the need to consider screening as a process of multiple steps rather than a one-step event. Completing the cascade of screening is critical to identifying those in need of treatment and a paramount precondition for effective treatment or elimination strategies. Second, the chances of identifying and linking patients with active HCV infection to treatment depend not only on test performances. Multistep screening processes are particularly prone to non-adherence effects and loss. Aligned with the fact that diagnostic tests indirectly affect outcomes but could directly affect pathways and clinical decisions, we stress the need that health economic studies for diagnostics should not only consider test performance aspects and population factors such as prevalence, but also take process related factors such as compliance or adherence into account. Third, test performance may vary substantially depending on the assays used. Scrutiny is required on tests used in practice before deriving conclusions from study results. Fourth, we propagate unique and consistent registration of patients not only along the cascade of care but also in the cascade of testing. Clear figures are required to particularly address drop-off issues or barriers to motivation preventing infected individuals from linkage to care and waste of resources accrued from incomplete diagnosis. Fifth, mapping the current testing practice revealed the need to involve a broad community of health care professionals. Rather than simply advocating a switch from one test to another in the laboratory, education and communication efforts are required. We regard the afore mentioned factors as critical for a successful implementation. Finally, our study suggests that there is a high probability that regardless of the uncertainty in AB performance, adherence rates or WTP, a streamlined approach employing a direct reflex test with AG on AB screening positive results would be a cost-effective alternative versus the current practice in Russia.

We acknowledge several limitations. Given the wide range of reasons that influence perception and drive motivation to adhere to recommended pathways and referrals, projections to other settings are generally hard to make and specific data should be subject to further research. The full implementation and use of a federal HCV registry is expected to provide more data and information helping to better design healthcare programs. There is uncertainty in the WTP threshold for detecting an infected subject which is not existing and was therefore tested over a wide range in sensitivity analyses. Reimbursement rates showed a high variability across Russia. The use of cost weighting factors may improve the accuracy of results once more specific data were available from different Russian regions. Albeit the cascade of testing in Russia requires people to move to various places, costs associated with people’s time, transportation, and loss of productivity have not been taking into account since these resources are beyond direct medical costs from a health system perspective. It should be noted however, that these consequences in multistep processes could be considerable, and likely impact people’s motivation to follow referrals. Considering these aspects would strengthen the need and preference for streamlined testing cascades. In general, findings from cost-effectiveness models depend on specific settings, and studies are needed to evaluate and compare the population-level benefits and costs of interventions, such as screening and treatment efforts, at each step. Considering long-term follow-up will add the value of missed infections on transmission, downstream comorbidities and costs. Within the concept of our study, related consequences were not considered but are likely to gravitate the preference to the most effective strategy. It is important to note that our model relies on the clinical condition, and takes in account the characteristics of different diagnostic tools in each of the stages of HCV infection. Also, it is the first study in this area that explicitly considered and discussed a patient-related dimension of the screening cascade (adherence) and its organizational impact (yield). Considering a loss to follow up is expected to present a much more realistic evaluation of strategies.

## Conclusion

Screening for hepatitis C in Russia is a complex process that involves several visits and stepwise testing, limiting adherence and substantially reducing the yield in the identification of active infections. Testing strategies with HCV antigen on HCV antibody positive cases offer a streamlining opportunity for population screening programs. These alternatives not only increased the case detection of active infections paramount for an effective linkage to treatment, they also improved the efficiency of screening by reducing visits and steps, and were found cost-effective compared to current practice in Russia. Non-adherence and loss to follow-up affects the performance of screening strategies particularly in multistep screening cascades and should be considered in health economic evaluations and before informing decisions.

## Supporting information

S1 File S1 File contains Table A. Abbreviations. Table B. Russian regions analyzed for reimbursement ratess. Table C. Input assumptions for base case and probabilistic scenario. Table D. Comparison of results from the deterministic base case analysis and PSA. Table E. Comparison of incremental results from the base case analysis and PSA. Table F. Cost-effectiveness results (Perfect adherence scenario). Table G. Proportion of incremental costs-effectiveness results per quadrant. Table H. Cost-effectiveness results (Base case scenario in US$). Fig A. HCV cascade of care in the Russian Federation. Fig B. False diagnosis in the perfect adherence scenario. Fig C. Incremental cost-effectiveness matrices. Fig D. One-way sensitivity analyses tornado diagrams. Fig E. One-way sensitivity analyses for seroprevalence and viremic rate. Fig F. Bivariate sensitivity analyses for adherence rates. Fig G. Bivariate sensitivity analyses for HCVAg testing costs vs. willingness-to-pay. Fig H. Bivariate sensitivity analyses for HCVAg testing costs vs. AG sensitivity in CHC. Fig I. Bivariate sensitivity analyses for HCVAb sensitivity vs. HCVAb specificity. Fig J. Expected value of perfect information vs. WPT.
(PDF)
