# Supplementary material for: Streamlining the screening cascade for active Hepatitis C in Russia: A cost-effectiveness analysis
Source: PLoS One. 2019 Jul 16;14(7):e0219687. doi: 10.1371/journal.pone.0219687 (PMC6634401; doi:10.1371/journal.pone.0219687)

File S1 Supporting Information

Table A. Abbreviations.

Table B. Russian regions analyzed for reimbursement rates.

Table C. Input assumptions for base case and probabilistic scenario.

Table D. Comparison of results from the deterministic base case analysis and PSA.

Table E. Comparison of incremental results from the base case analysis and PSA.

Table F. Cost-effectiveness results (Perfect adherence scenario).

Table G. Proportion of incremental costs-effectiveness results per quadrant.

Table H. Cost-effectiveness results (Base case scenario in US$).

Fig A. HCV cascade of care in the Russian Federation.

Fig B. False diagnosis in the perfect adherence scenario.

Fig C. Incremental cost-effectiveness matrices.

Fig D. One-way sensitivity analyses tornado diagrams.

Fig E. One-way sensitivity analyses for seroprevalence and viremic rate.

Fig F. Bivariate sensitivity analyses for adherence rates.

Fig G. Bivariate sensitivity analyses for HCVAg testing costs vs. willingness-to-pay.

Fig H. Bivariate sensitivity analyses for HCVAg testing costs vs. AG sensitivity in CHC.

Fig I. Bivariate sensitivity analyses for HCVAb sensitivity vs. HCVAb specificity.

Fig J. Expected value of perfect information vs. WPT.

Table A. Abbreviations.

AB	Laboratory test: Anti-HCV antibody test
AG	Laboratory test: HCV antigen test
AHC	Infection status: Acute HCV infection
AI	Diagnosis: Active infection
CHC	Infection status: Chronic HCV infection
CPDI	Result category: Costs per diagnosed infection ratio
DAA	Direct acting antivirals
DAI	Result category: Diagnosed active HCV infection
HCV	Hepatitis C virus
ICER	Result category: Incremental cost-effectiveness ratio
INMB	Result category: Incremental net monetary benefit
MAI	Result category: Missed active HCV infection
NMB	Net monetary benefit
PSA	Probabilistic sensitivity analysis
RNA	Laboratory test: Test for HCV RNA by nucleic acid amplification methods
SVR	Sustained virological response
UE	Diagnosis: Unexposed to HCV
UI	Diagnosis: Not infected with HCV

Table B. Russian regions analyzed for reimbursement rates.

**Region**	**Federal district**
Tver region	Central
Kaliningrad	North West
Volgograd region	South
Dagestan	North-Caucasian
Orenburg	Volga
Primoskiy region	Ural
Khanty Mansi Autonomous Area	Sibir
Kemerovo region	Far East

Table C. Input assumptions from base case and probabilistic scenario.

Variable	Basecase	PSA distribution output, n=5,000						
		Mean	StDev	Minimum	Q1	Median	Q3	Maximum
Prevalence AHC	0.000015	0.000015	0	0.000014	0.000015	0.000015	0.000015	0.000016
Prevalence AB	0.041	0.041	0.002	0.034	0.040	0.041	0.042	0.048
Viremic rate	0.71	0.710	0.014	0.660	0.701	0.710	0.720	0.767
AG sensitivity (AHC)	0.999	0.999	0.001	0.989	0.999	0.999	1.000	1.000
AG sensitivity (CHC)	0.967	0.967	0.001	0.964	0.967	0.967	0.968	0.970
AG specificity	0.990	0.990	0.001	0.986	0.990	0.990	0.991	0.994
AB sensitivity (AHC)	0.600	0.600	0.003	0.590	0.598	0.600	0.602	0.611
AB sensitivity (CHC)	0.818	0.818	0.003	0.807	0.816	0.818	0.820	0.829
AB specficity	0.997	0.997	0.001	0.993	0.996	0.997	0.998	0.999
RNA sensitivity	0.9990	0.0005	0.9953	0.9987	0.9991	0.9994	1.0000	0.9990
RNA specificity	0.9990	0.0005	0.9959	0.9987	0.9991	0.9994	0.9999	0.9990
Adherence rate A1	0.95	0.95	0.02	0.84	0.94	0.95	0.97	0.99
Adherence rate A2	0.95	0.95	0.02	0.79	0.94	0.95	0.96	0.99
Adherence rate A3	0.75	0.75	0.02	0.68	0.74	0.75	0.76	0.82
Adherence rate A4	0.8	0.80	0.02	0.71	0.79	0.80	0.81	0.87
Adherence rate A5	0.95	0.95	0.02	0.83	0.94	0.95	0.96	1.00
Adherence rate A6	0.95	0.95	0.02	0.86	0.94	0.95	0.96	0.99
Adherence rate A7	0.5	0.50	0.02	0.41	0.49	0.50	0.51	0.59
Costs: AB	252	210	82	101	140	192	270	405
Costs: AG	600	601	197	158	459	570	708	1750
Costs: RNA	1339	1113	417	342	783	1067	1419	2027
Costs: Visit	338	338	26	258	320	337	354	432
Costs: Genotyping	1841	1745	458	1078	1354	1675	2094	2763
Costs: Blood draw	123	119	31	74	93	114	142	190
Costs: Ultrasound	519	399	205	152	233	341	523	948
Costs: Clin. tests	1028	762	198	455	589	736	920	1169

All costs in 2017 Rubles (100 RUB = USD 4.11). Q1 and Q3 refer to the 25th and 75th percentile of data. Parameters α, β for beta distributions were parameterized from the mean and standard deviation as described in Table 1 by $\alpha = \text{mean} * \left(\frac{mean(1-mean)}{SD^2} - 1\right), \beta = (1-mean)(\frac{mean}{SD^2}(1-mean) - 1)$. For lognormal distributions, parameters μ, σ were estimated from the mean and median as shown in Table 1 by $\mu = \ln(median), \sigma = \sqrt{2ln\frac{(mean)}{(median)}}$.

Table D. Comparison of results from the deterministic base case analysis and PSA.

Outcome		DiaAcc, %	Yield, %	DAI	Costs	CPDI	NMB
Strategy I	**Base case**	99.387	88.99	1,184	1,114	94,121	-1,114
	**Mean**	99.387	88.95	1,181	1,053	89,599	-1,030
	**95%CI**	(99.386; 99.388)	(88.88; 89.03)	(1,179; 1,184)	(1,050; 1,056)	(71,435; 112,349)	(-1,033; -1,027)
	**Median**	99.388	89.18	1,180	1,043	88,700	-1,019
	**(Q1; Q3)**	(99.365; 99.411)	(87.31; 90.86)	(1,120; 1,239)	(975; 1,125)	(81,974; 96,402)	(-1,101; -951)
Strategy II	**Base case**	99.456	89.40	2,002	1,115	55,704	-1,115
	**Mean**	99.456	89.37	2,001	1,058	53,072	-1,018
	**95%CI**	(99.455; 99.457)	(89.29; 89.45)	(1,997; 2,004)	(1,056; 1,061)	(43,211; 65,101)	(-1,021; -1,016)
	**Median**	99.456	89.62	1,999	1,049	52,582	-1,009
	**(Q1; Q3)**	(99.437; 99.477)	(87.74; 91.29)	(1,911; 2,089)	(985; 1,125)	(48,968; 56,743)	(-1,085; -945)
Strategy III	**Base case**	99.379	90.15	1,975	1,097	55,522	-1,097
	**Mean**	99.379	90.11	1,974	1,043	53,004	-1,003
	**95%CI**	(99.378; 99.389)	(90.03; 90.19)	(1,971; 1,978)	(1,040; 1,046)	(43,035; 65,067)	(-1,006; -1,001)
	**Median**	99.379	90.34	1,973	1,034	52,520	-995
	**(Q1; Q3)**	(99.357; 99.402)	(88.47; 92.05)	(1,886; 2,061)	(969; 1,109)	(48,872; 56,700)	(-1,069; -929)
Strategy IV	**Base case**	98.963	90.08	2,415	1,454	60,202	-1,454
	**Mean**	98.964	90.05	2,414	1,435	59,633	-1,386
	**95%CI**	(98.961; 98.966)	(89.97; 90.12)	(2,410; 2,418)	(1,429; 1,440)	(45,682; 79,867)	(-1,392; -1,381)
	**Median**	98.970	90.27	2,411	1,410	58,470	-1,362
	**(Q1; Q3)**	(98.902; 99.03)	(88.41; 91.98)	(2,305; 2,521)	(1,295; 1,544)	(53,481; 64,674)	(-1,497; -1,246)

Diagnostic accuracy (DiaAcc) evaluated under the assumption of perfect adherence. Mean, median and confidence interval from probability sensitivity analysis (PSA) with 5,000 runs. DAI Diagnosed active infections per 100,000 tested. NMB net monetary benefit assuming a WTP of 2,000 Rubles. CPDI: Cost per diagnosed infection ratio. Confidence intervals for CPDI and NMB estimated from the 2.5th and 97.5th percentile of the respective result distribution.

Table E. Comparison of incremental results from the base case analysis and PSA.

Incremental outcome		Incr. DiaAcc, %	Incr. yield, %	Incr. DAI	Incr. costs	ICER	INMB
**Strat (II) - Strat (I)**	**Base case**	0.069	0.41	819	1	155	15
	**Mean**	0.069	0.41	820	5	585	11
	**95%CI**	(0.067; 0.070)	(0.31; 0.52)	(815; 824)	(1; 9)	(-2,560; 3,698)	(-14 to 36)
**Strat (III) - Strat (I)**	**Base case**	-0.008	1.15	792	-17	-2,176	33
	**Mean**	-0.008	1.16	793	-11	-1,373	26
	**95%CI**	(-0.010; -0.007)	(1.05; 1.26)	(789; 797)	(-15; -7)	(-5,011; 2,033)	(0 to 54)
**Strat (IV) - Strat (I)**	**Base case**	-0.425	1.09	1,232	340	27,606	-315
	**Mean**	-0.424	1.09	1,233	381	31,117	-357
	**95%CI**	(-0.427; -0.421)	(0.98; 1.20)	(1,228; 1,238)	(375; 388)	(3,337; 69,512)	(-826 to -16)
**Strat (II) - Strat (III)**	**Base case**	0.077	-0.74	27	19	69,136	-18
	**Mean**	0.077	-0.74	27	16	58,227	-15
	**95%CI**	(0.076; 0.078)	(-0.85; -0.63)	(22; 32)	(12; 19)	(40,541; 80,505)	(-21 to -10)
**Strat (IV) - Strat (II)**	**Base case**	-0.493	0.68	413	339	82,002	-330
	**Mean**	-0.492	0.68	413	376	91,463	-368
	**95%CI**	(-0.493; -0.490)	(0.57; 0.79)	(407; 419)	(370; 382)	(13,678; 201,226)	(-817 to -48)

Diagnostic accuracy (DiaAcc) evaluated under the assumption of perfect adherence. Mean and confidence intervals from probability sensitivity analysis (PSA) with 5,000 runs. DAI Diagnosed active infections per 100,000 tested. All costs in 2017 RUB (100 RUB = $4.11). NMB: net monetary benefit assuming a WTP of 2,000 Rubles ($82). ICER: Incremental cost-effectiveness ratio. INMB: Incremental net monetary benefit. Confidence intervals for ICER, and INMB estimated from the 2.5th and 97.5th percentile of the respective result distribution.

Table F. Cost-effectiveness results (Perfect adherence scenario).

	Costs, RUB			Diagnosed active infections (DAI)			CPDI		ICER		INMB	
**Strategy**	**Mean**	**Incr.**	**Incr. 95%CI**	**Mean**	**Incr.**	**Incre. 95%CI**	**Mean**	**95%CI***	**Mean**	**95%CI***	**Mean**	**95%CI***
Strategy I	1,169			2,300			50,975	(41,675; 62,959)				
**Strategy III**	**1,105**	**-65**	**(-69; -60)**	**2,303**	**3**	**(-2; 8)**	48,092	**(39,231; 59,256)**	**-167,1891**	**(-21,482,311; 16,254,354)**	**65**	**(31; 102)**
Strategy II	1,147	42	(-381; -369)	2,382	78	(73; 83)	48,294	(39,507; 59,168)	54,289	(37,197; 75,219)	-41	(-56; -28)
Strategy IV	1,522	375	(411; 424)	2,816	435	(429; 441)	54,193	(41,564; 71,997)	86,529	(6,584; 196,471)	-366	(-842; -669)

Results from Monte Carlo simulation with 5,000 iterations (PSA). List sorted by increasing number of diagnosed active infections. All costs in 2017 Rubles (100 RUB = $4.11). DAI: Diagnosed active HCV infection per 100,000 screened subjects. CPDI: Costs per diagnosed active infection. ICER: Incremental cost-effectiveness ratio; incremental costs divided by incremental DAI. INMB: Incremental Net Monetary Benefit. CPDI, ICER, and INMB confidence intervals estimated from the 2.5th and 97.5th percentile of the respective result distributions. The most cost-effective strategy at a WTP threshold of 2,000 Rubles per additional detected infection is shown in bold.

Table G. Proportion of incremental cost-effectiveness results per quadrant.

COMPONENT	QUADRANT	INCREFF	INCRCOST	INCRCE	II vs. I	III vs. I	IV vs. I	II vs. III	IV vs. II
C1	IV	IE>0	IC<0	Superior	0.35	0.77	0.01	0.00	0.01
C2	I	IE>0	IC>0	ICER<WTP	0.46	0.20	0.01	0.00	0.00
C3	III	IE<0	IC<0	ICER>WTP	0.00	0.00	0.00	0.00	0.00
C4	I	IE>0	IC>0	ICER>WTP	0.19	0.03	0.98	1.00	0.99
C5	III	IE<0	IC<0	ICER<WTP	0.00	0.00	0.00	0.00	0.00
C6	II	IE<0	IC>0	Inferior	0.00	0.00	0.00	0.00	0.00
Indiff	origin	IE=0	IC=0	0/0	0.00	0.00	0.00	0.00	0.00

Probabilistic sensitivity analysis with 5,000 iterations. Willingness-to-pay (WTP) threshold 2,000 Rubles.

Table H. Cost-effectiveness results (Base case scenario in US$).

	Costs, US$			DAI			CPDI		ICER	
**Strategy**	**Mean**	**Incr.**	**Incr. 95%CI**	**Mean**	**Incr.**	**Incre. 95%CI**	**Mean**	**95%CI***	**Mean**	**95%CI***
Strategy I	43.27			1181			3681	(2935; 4615)		
**Strategy III**	**42.84**	**-0.43**	**(-0.60; -0.27)**	**1974**	**793**	**(789; 797)**	**2178**	**(1775; 2674)**	**-56**	**(-206; 83)**
Strategy II	43.48	0.64	(0.48; 0.80)	2001	27	(22; 32)	2180	(1768; 2673)	2392	(1665; 3307)
Strategy IV	58.93	15.46	(15.20; 15.71)	2414	413	(407; 419)	2450	(1877; 3281)	3757	(562; 8267)

Results from Monte Carlo simulation with 5,000 iterations (PSA). List sorted by increasing number of diagnosed active infections. All costs calculated in 2017 Rubles and converted to US$ (100 RUB = $4.11). DAI: Diagnosed active HCV infection per 100,000 screened subjects. CPDI: Costs per diagnosed active infection. ICER: Incremental cost-effectiveness ratio; incremental costs divided by incremental DAI. CPDI and ICER confidence intervals estimated from the 2.5th and 97.5th percentile of the respective result distributions. The most cost-effective strategy at a WTP threshold of $82 per additional detected infection is shown in bold.

Fig A. HCV cascade of care in the Russian Federation.

Estimated number of subjects positive for HCV antibodies (estimated prevalence), and number of patients in each step of the cascade of care as derived from the Russian Registry of chronic HCV. AB: Anti-HCV antibody; RNA: HCV nucleic acid; SVR: Sustained virological response.

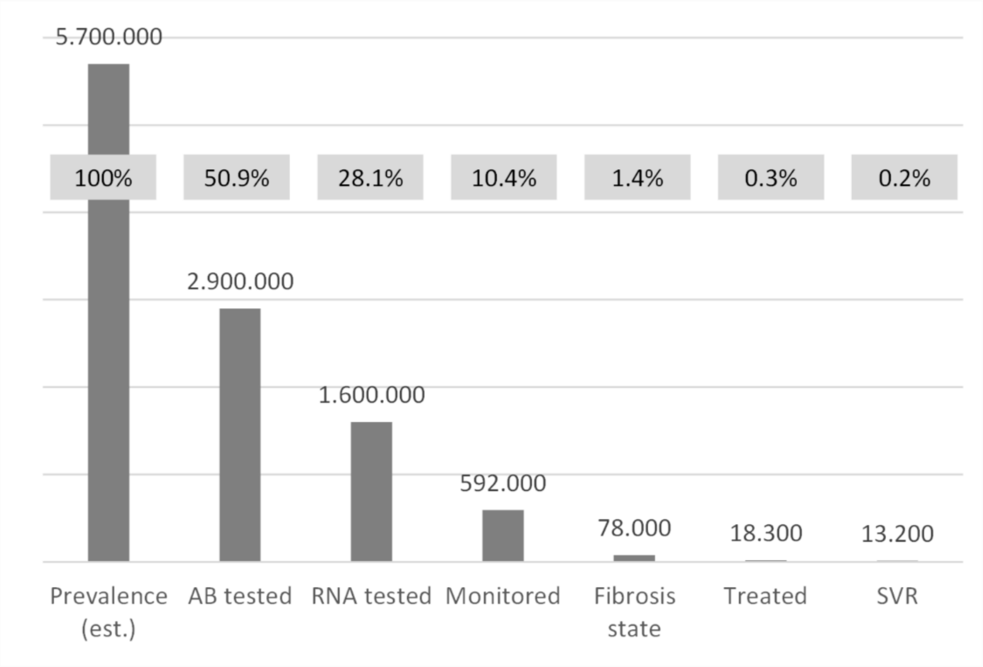

Fig B. Number of false diagnosis in the perfect adherence scenario.

Number of false positive (FPos) and false negative (FNeg) diagnosis per 100,000 people screened assuming perfect adherence to the required steps in the cascade of testing. Strategy-I (I), Strategy-II (II), Strategy-III (III), Strategy-IV (IV).

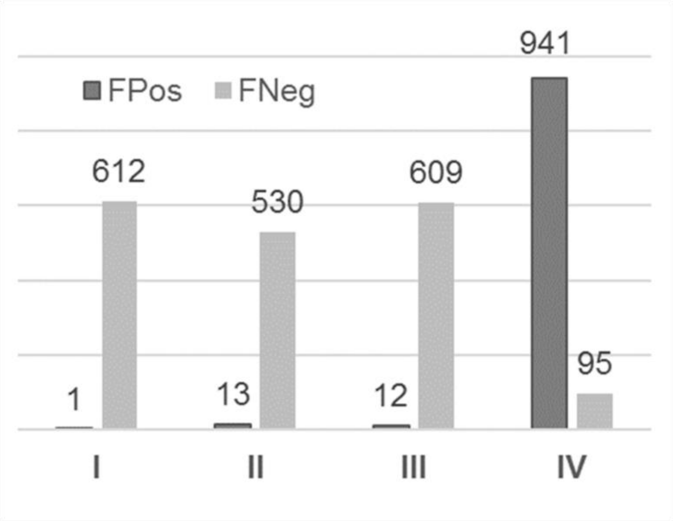

Fig C. Incremental cost-effectiveness matrices.

Incremental number of diagnosed infections and costs comparing different screening cascades. A: Strategy II vs. Strategy I, B: Strategy III vs. Strategy I, C: Strategy II vs. Strategy III, and D: Strategy IV vs. Strategy II. Each point in the cloud corresponds to a result of one out of 5,000 iterations of the probabilistic sensitivity analysis. Dark curves indicate the 95% confidence interval. Costs in 2017 Rubles (100 RUB = $4.11). The dotted lines reflect the WTP threshold at 2,000 Rubel ($82).

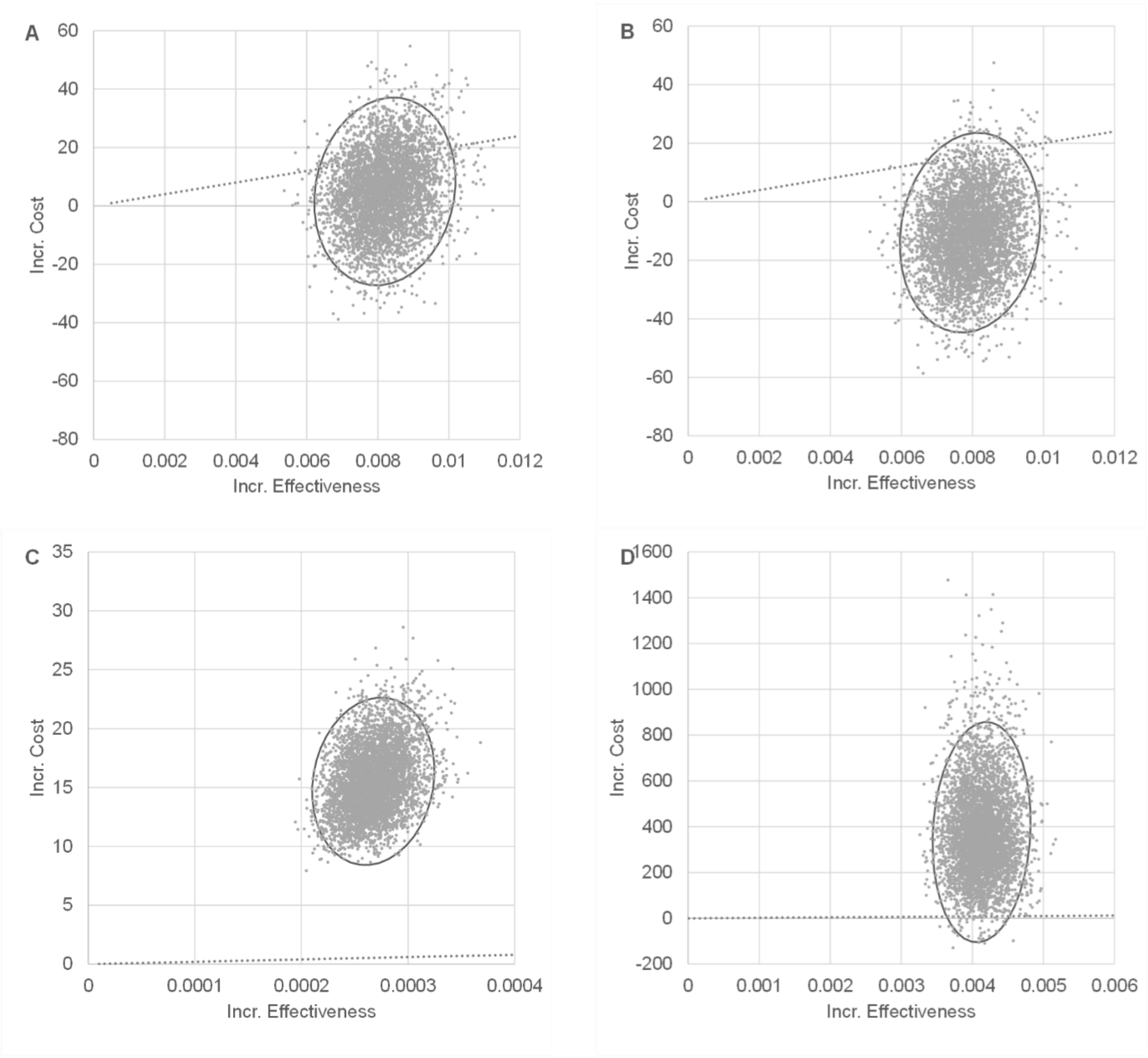

Fig D. One-way sensitivity analyses tornado diagrams.

Incremental cost-effectiveness ratio tornado diagrams from multiple one-way sensitivity analyses on all variables within the ranges stated in brackets. ICER: Incremental costs divided by incremental number of diagnosed active infections between two strategies. A: Strategy II vs. Strategy I, B: III vs. IV, C: II vs. III, and D: V vs. II. The base case is represented by the vertical line. Costs in 2017 Rubles (100 RUB = $4.11). The willingness-to-pay threshold at 2,000RUB ($82) is indicated by the dotted line. Variables not presented here did not show an effect within the tested ranges.

A

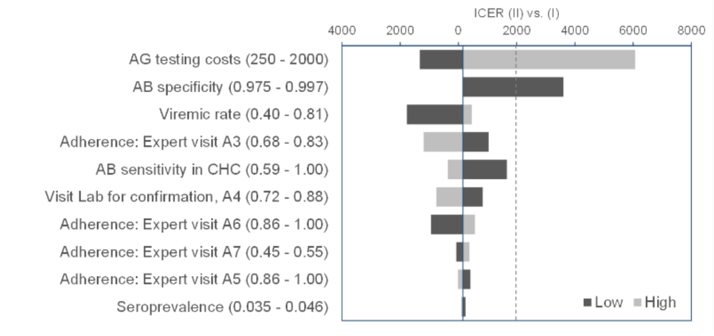

B

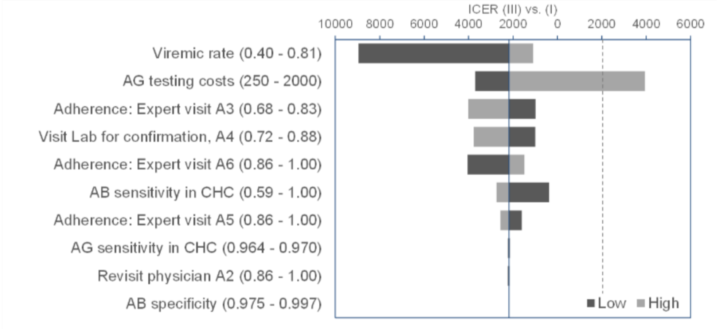

C

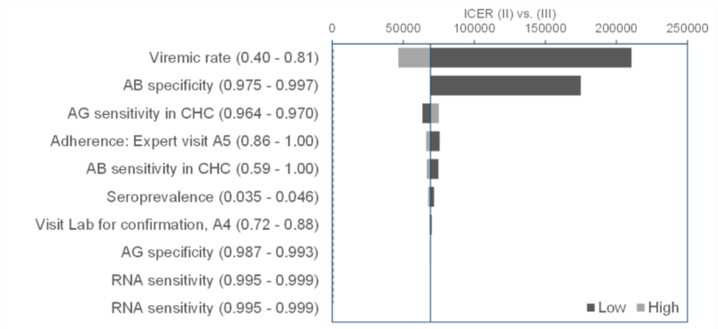

D

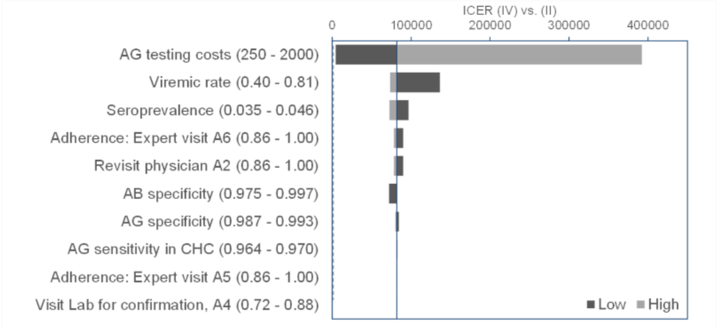

Fig E. One-way sensitivity analyses for seroprevalence and viremic rate.

Results represented as net monetary benefit (NMB). The dotted lines reflect the base case (BC) assumption. Costs based on 2017 Rubel (100 RUB = $4.11) NMB calculations assuming a WTP of 2,000 Rubel ($82). The preferred strategy is indicated by the highest NMB. Strategy I (S-I); Strategy II (S-II); Strategy III (S-III); Strategy IV (S-IV).

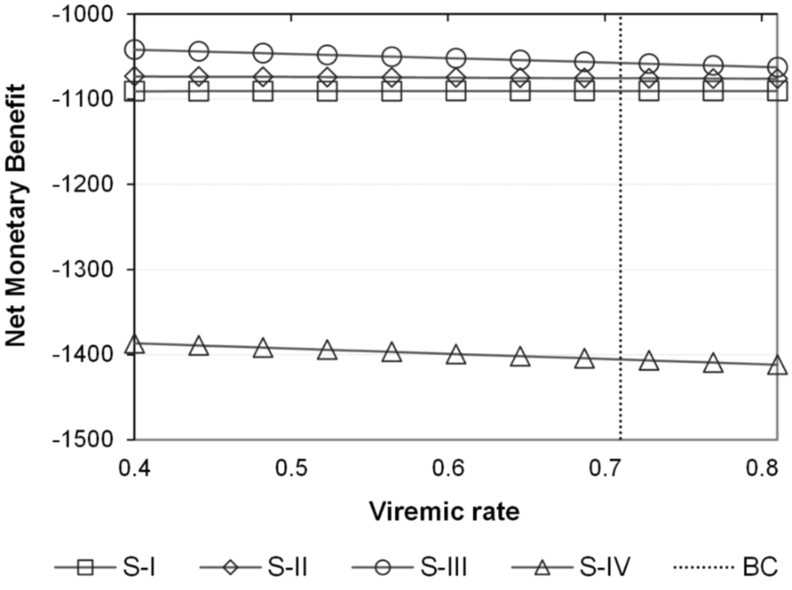

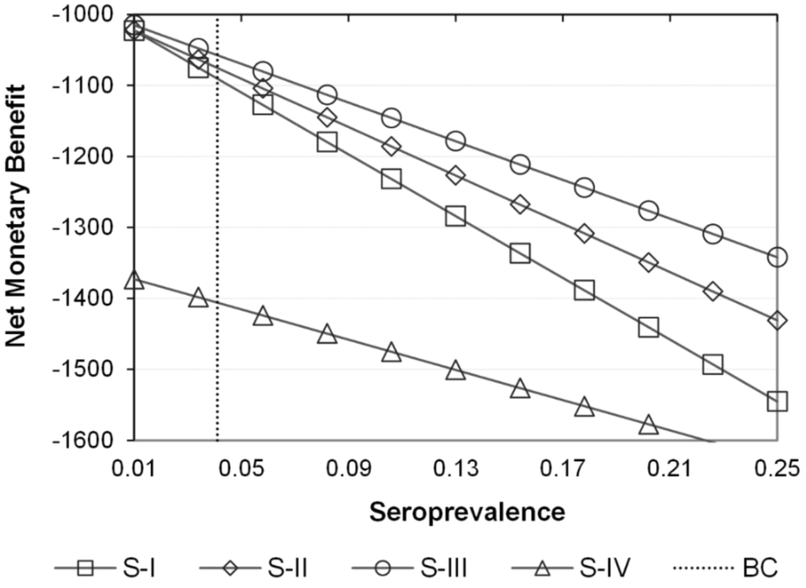

Fig F. Bivariate sensitivity analyses for adherence rates.

Bivariate threshold analysis for Strategy-II (S-II), Strategy-III (S-III), and Strategy-IV (S-IV) vs. the Standard (S-I). BC: Base case assumption. Preferred strategy in terms of net monetary benefit (NMB) based on costs in 2017 Rubles (100 Rub = $4.11) and assuming a willingness-to-pay of 2,000 Rubles ($82) per diagnosed active infection.

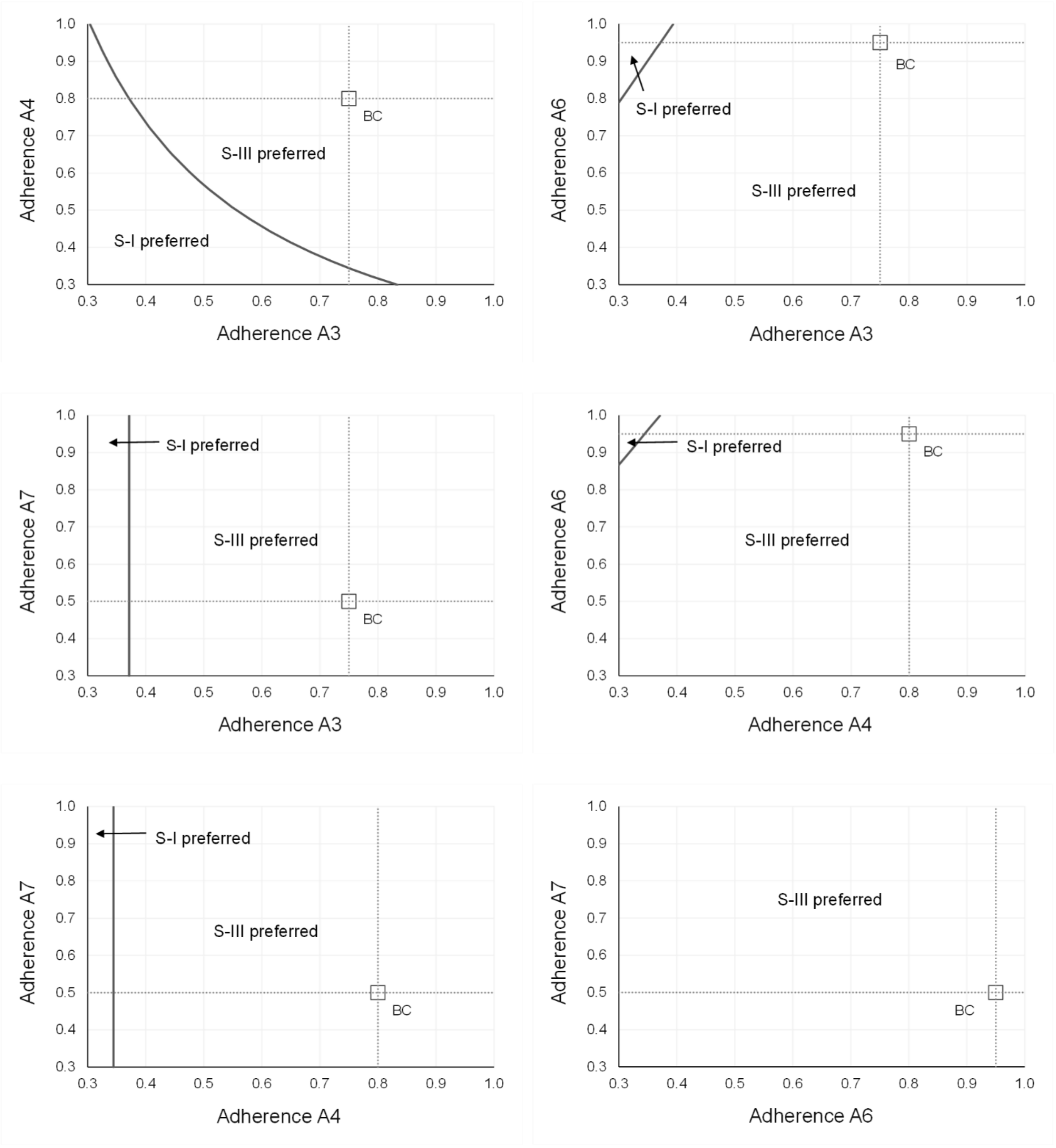

Fig G. Bivariate sensitivity analyses for HCVAg testing costs vs. willingness-to-pay.

Bivariate threshold analysis for Strategy-II (S-II), Strategy-III (S-III), and Strategy-IV (S-IV) vs. the Standard (S-I). BC: Base case assumption. At the threshold line, the respective alternative strategy was found equivalent to the S-I in terms of net monetary benefit (NMB) based on costs in 2017 Rubles (100 RUB = $4.11) and assuming a willingness-to-pay of 2,000 Rubles ($82) per diagnosed active infection. Below the threshold line, the alternative strategy was superior to S-I.

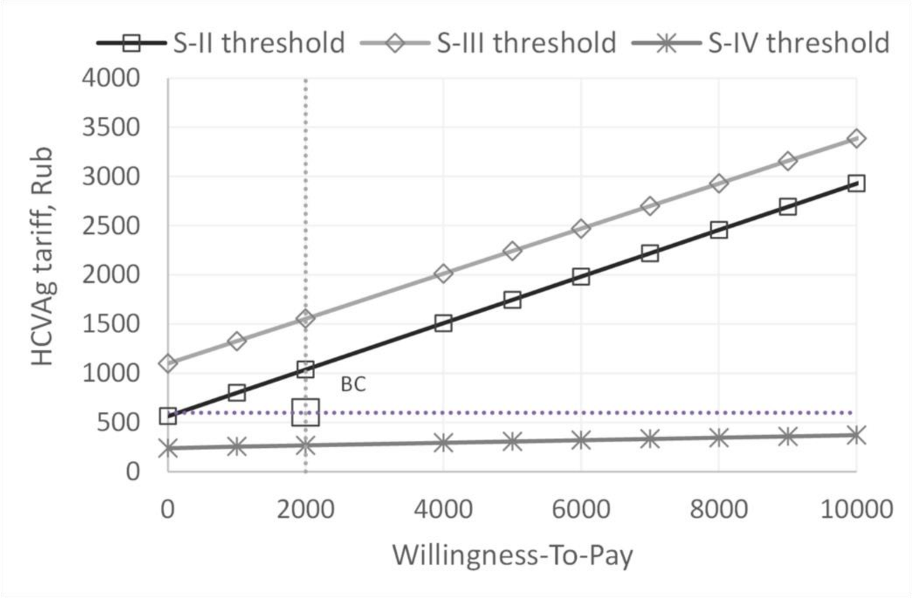

Fig H. Bivariate sensitivity analyses for HCVAg testing costs vs. AG sensitivity in CHC.

Preferred strategy in terms of net monetary benefit (NMB) based on costs in 2017 Rubles (100 Rub = $4.11) and assuming a willingness-to-pay of 2,000 Rubles ($82) per diagnosed active infection. At the threshold line, strategies were found equivalent in terms of NMB. BC: Base case assumption. S-I: Strategy-I; S-III: Strategy-III. Strategies S-II and S-IV were not preferred over the tested ranges.

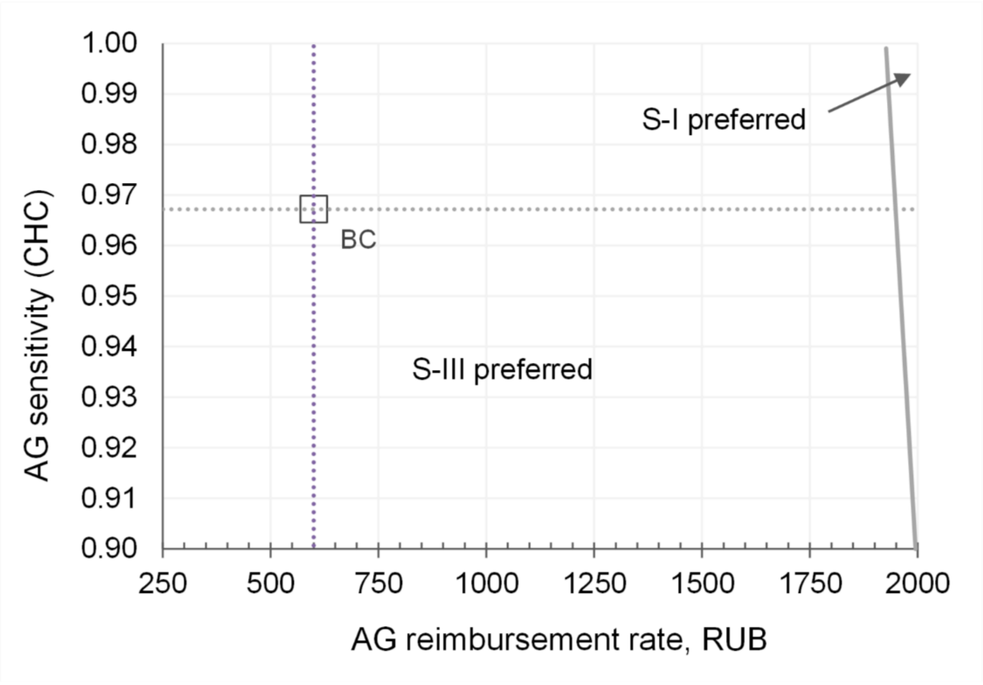

Fig I. Bivariate sensitivity analyses for HCVAb sensitivity vs. HCVAb specificity.

Bivariate threshold analysis for Strategy-II (S-II), Strategy-III (S-III), and Strategy-IV (S-IV) vs. the Standard (S-I). Preferred strategy in terms of net monetary benefit (NMB) based on costs in 2017 Rubles (100 Rub = $4.11) and assuming a willingness-to-pay of 2,000 Rubles ($82) per diagnosed active infection. BC: Base case assumption. At the threshold line, the respective alternative strategy was found equivalent to the standard in terms of NMB. No threshold line appears in the graph for S-III and S-IV, with S-III being superior to S-I, and S-IV being inferior to S-I across the tested ranges.

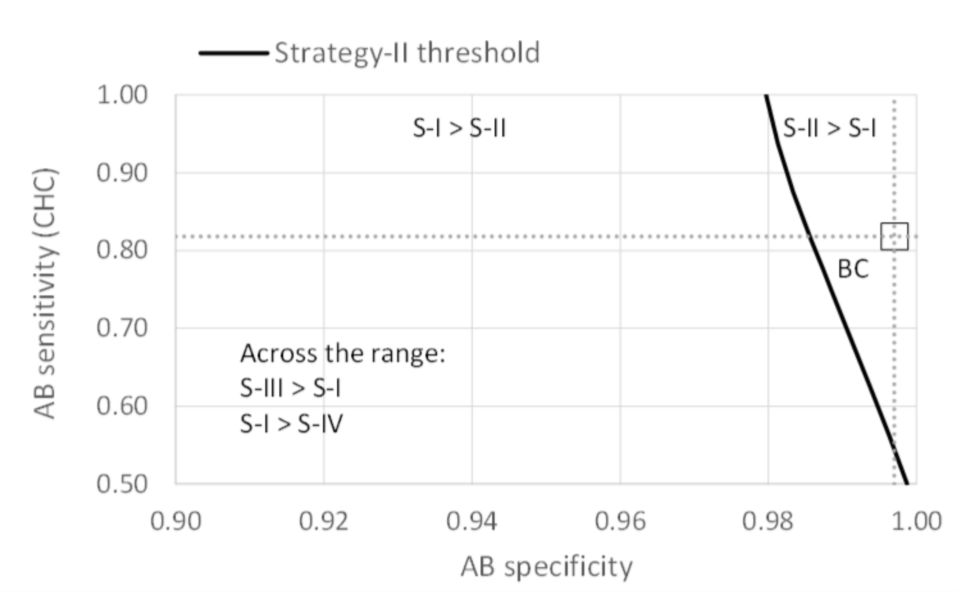

Fig J. Expected value of perfect information vs. WPT.

Overall mean expected value of perfect information (EVPI) for different willingness-to-pay (WTP) thresholds per diagnosed active infections (DAI) for estimating the costs of existing uncertainty. WTP in 2017 Rubles (100 RUB = $4.11). The base case scenario was assuming a WTP of 2,000 RUB ($82).

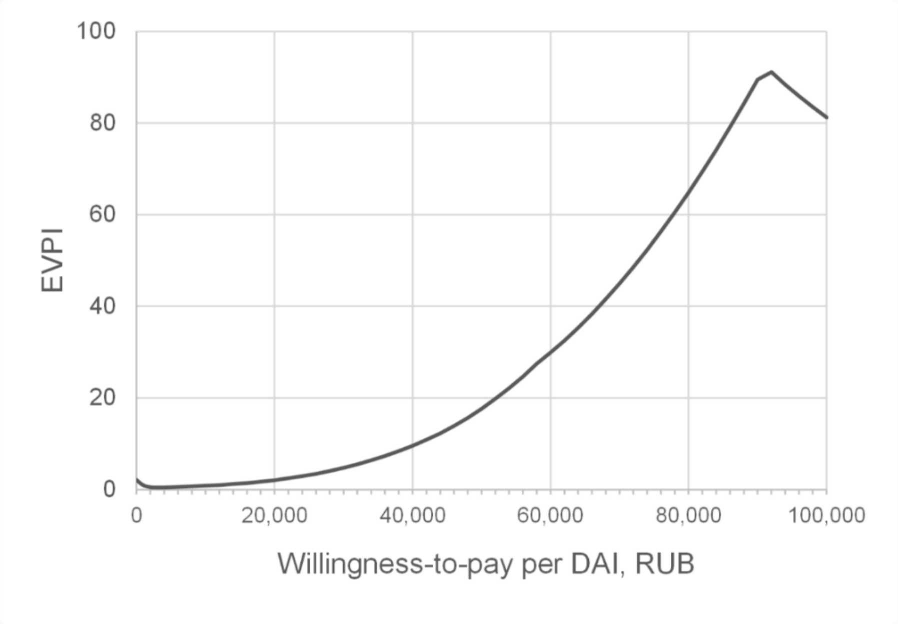

Supplement: S1 File — contains Table A. Abbreviations. Table B. Russian regions analyzed for reimbursement ratess. Table C. Input assumptions for base case and probabilistic scenario. Table D. Comparison of results from the deterministic base case analysis and PSA. Table E. Comparison of incremental results from the base case analysis and PSA. Table F. Cost-effectiveness results (Perfect adherence scenario). Table G. Proportion of incremental costs-effectiveness results per quadrant. Table H. Cost-effectiveness results (Base case scenario in US$). Fig A. HCV cascade of care in the Russian Federation. Fig B. False diagnosis in the perfect adherence scenario. Fig C. Incremental cost-effectiveness matrices. Fig D. One-way sensitivity analyses tornado diagrams. Fig E. One-way sensitivity analyses for seroprevalence and viremic rate. Fig F. Bivariate sensitivity analyses for adherence rates. Fig G. Bivariate sensitivity analyses for HCVAg testing costs vs. willingness-to-pay. Fig H. Bivariate sensitivity analyses for HCVAg testing costs vs. AG sensitivity in CHC. Fig I. Bivariate sensitivity analyses for HCVAb sensitivity vs. HCVAb specificity. Fig J. Expected value of perfect information vs. WPT. (PDF) [file pone.0219687.s001.pdf]
